# The ‘welcomed lockdown’ hypothesis? Mental wellbeing and mobility restrictions

**DOI:** 10.1007/s10198-022-01490-6

**Published:** 2022-08-12

**Authors:** Joan Costa-Font, Martin Knapp, Cristina Vilaplana-Prieto

**Affiliations:** 1 Department of Health Policy, London School of Economics and Political Science (LSE), CESIFo & IZA, London, UK; 2 Department of Health Policy, London School of Economics and Political Science, London, UK; 3 University of Murcia, Murcia, Spain

**Keywords:** Anxiety, Depression, COVID-19, Pandemic, Lockdown, 118

## Abstract

The COVID-19 pandemic and its mobility restrictions have been an external shock, influencing mental wellbeing. However, does risk exposure to COVID-19 affect the mental wellbeing effect of lockdowns? This paper examines the ‘welcomed lockdown’ hypothesis, namely the extent to which there is a level of risk where mobility restrictions are not a hindrance to mental wellbeing. We exploit the differential timing of exposure the pandemic, and the different stringency of lockdown policies across European countries and we focus on the effects on two mental health conditions, namely anxiety and depression. We study whether differences in the individual symptoms of anxiety and depression are explained by the combination of pandemic mortality and stringency of lockdown. We draw on an event study approach, complemented with a Difference-in-Difference (DiD), and Regression Discontinuity Design (RDD). Our estimates suggest an average increase in depression (3.95%) and anxiety (10%) symptoms relative to the mean level on the day that lockdown took effect. However, such effects are wiped out when a country’s exhibits high mortality (‘pandemic category 5’). Hence, we conclude that in an environment of high mortality, lockdowns no longer give rise to a reduction in mental wellbeing consistent with the ‘welcome lockdown’ hypothesis.

**Supplementary Information:**

The online version contains supplementary material available at 10.1007/s10198-022-01490-6.

## Introduction

Pandemics can exert important detrimental effects on individuals’ mental wellbeing. The risk of contagion can trigger anxiety and depressive symptoms. However, these effects are only partly the direct result of exposure to COVID-19 risk (in this case, the risk of infection), but also result from the stringency of policy restrictions. Policy restrictions include spatial lockdowns alongside a number of regulatory measures that restrict individual freedoms, and impose other duties such as the obligation to wear face masks in public places, the need for social distancing, the implementation of temperature checks, and the use of hand gels. Each of these measures can protect against the risks of infection, but, at the same time, they act as *reminders of the severity of the pandemic*. This paper examines the effect of policy restrictions and different levels of  pandemic severity on mental wellbeing using evidence from the first wave of COVID-19 pandemic.

Unlike previous pandemics, COVID-19 has spread at an unprecedented speed, especially in European countries, which had barely a few weeks to react. Individuals could not learn from previous pandemics as such outbreaks took place overseas—mostly in East Asian countries. However, policy measures that have been put in place to fight COVID-19 have been heterogeneous across European countries, which provide rich quasi-experiment variation to examine the effect of different policy stringency measures on mental wellbeing. We can identify the combined effect of pandemic restrictions and risk exposure during the first wave of COVID-19, as infection numbers have been recorded and communicated to the general population when outbreaks have occurred.^1^

Previous studies have already documented detrimental mental health effects of COVID-19 and policy restrictions. Banks and Xu [4, 6] find a deterioration of mental wellbeing among those who had a mental disorder prior to COVID-19, though other studies exploring the effects of lockdowns find evidence of a rise in mental distress compared to pre-pandemic levels [20, 66]. However, previous studies are very much country-specific and do not consider the effect of risk exposure combined with policy restrictions. We attempt to fill this gap. 

**Fig. 1:**
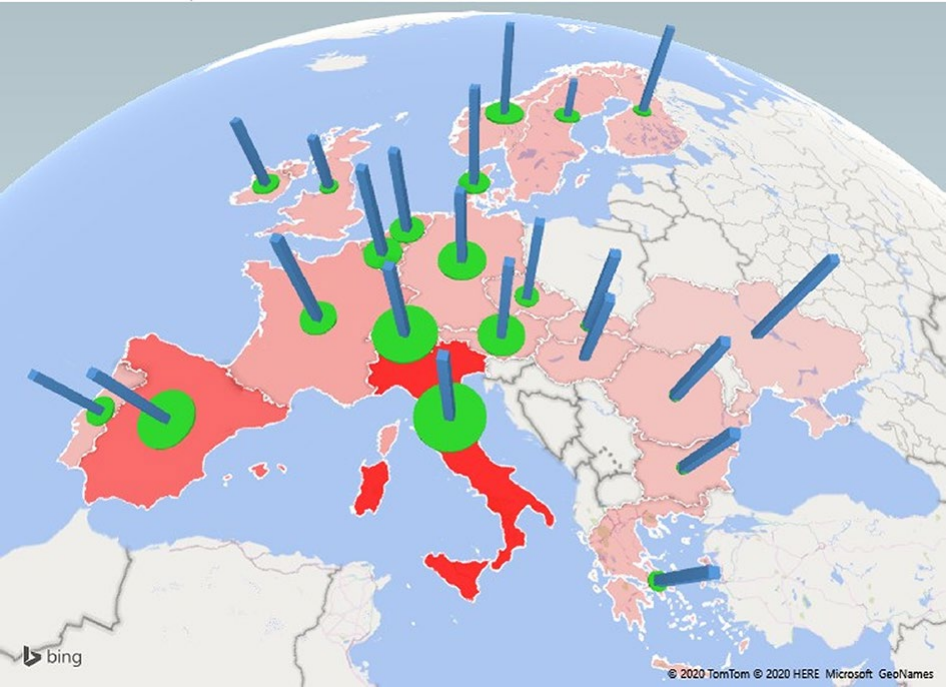
Stringency Index (blue bricks) and risk exposure (green circles) and deaths per million (red areas). Date: March 20, 2020. Red areas correspond to the mortality rate (deaths per 1,000,000 inhabitants). Higher color intensity denotes higher mortality rate. Data come from https://ourworldindata.org/coronavirus-data-explorer. Green circles correspond to the exposure rate to COVID-19 (confirmed cases per 1,000,000 inhabitants). Larger diameter denotes higher exposure to the virus. Data come from https://ourworldindata.org/coronavirus-data-explorer. Blue Bricks corresponds to for COVID-19 Government Response Stringency Index (Stringency Index). Higher height denotes higher stringency. Data come from https://www.bsg.ox.ac.uk/research/research-projects/oxford-COVID-19-government-response-tracker

Figure 1 combines evidence of exposure to risk and stringency of government responses in 22 European countries on March 20th, 2020.^2^ In almost all countries (with the noticeable fundamental exception of Sweden and the United Kingdom), the value of the Stringency Index (a composite measure based on indicators including prohibition of public meetings, total or partial school closures and workplace closures and introduction of travel restrictions bans within and between countries; see below) is large. Such aggregate results suggest that the spread of the pandemic and the associated mortality rates differ widely between countries.

This paper studies the combined effect of risk exposure and policy restrictions to specifically empirically test the so-called ‘welcomed lockdown’ hypothesis, namely the extent to which there is a level of risk where mobility restrictions are not a hindrance to mental wellbeing. That is, we examine the mental wellbeing effects of mobility restrictions resulting from COVID-19 together with risk exposure (proxied by COVID-19 fatality rates). We examine whether differences in symptoms of anxiety and depression are explained by differences in mortality and stringency of lockdown measures using several strategies. One of those strategies includes the Coarsened Exact Matching (CEM). The CEM is an innovative matching methodology developed by Iacus et al. [38]. Previous work in the literature addressing similar research questions has attempted to address these identification problems by relying on propensity score matching [43]^3^. We exploit data from a European sub-sample (22 countries) retrieved from an online survey conducted globally between March 20th and April 6th 2020, which implied that the selection of the counterfactual is the crucial step for the correct quantification of the average treatment effect.

To our knowledge, this is the first study to use CEM to estimate the causal effect of the imposition of lockdowns due to the COVID-19 pandemic on mental health. Our empirical strategy includes an event study followed by a Difference-in-Difference (DiD) with and without a Regression Discontinuity (RD) design.

On the day that lockdown took effect, we estimate an increase in depression (2.76%) and anxiety (7.40%) symptoms relative to the mean level. However, the interaction of lockdown with high  COVID-19 mortality (pandemic category 5) results in a considerable reduction in the symptoms of depression (−6.46%) and anxiety (−8.43%). Secondly, although the announcement of a pandemic as ‘level 5’ gaves rise to an increase in the symptoms of depression (2.16%) and anxiety (13.80%), a lockdown call in this context actually reduced anxiety by almost 20%.

## Related literature

### Previous pandemics

Evidence from several pandemics and epidemics across the world suggests consistent evidence that they exert a detrimental impact on mental health, in some cases such effects are long-lasting. Individuals infected with SARS (Severe Acute Respiratory Syndrome) in Hong Kong exhibited a rise in moderate and severe mental disorders, such as anxiety and depression [19]. Similarly, Kim et al. [42] found that 70% of patients hospitalized for MERS (Middle East Respiratory Syndrome) in South Korea experienced a mental disorder while hospitalized, but 40% of those who were infected continued to use psychiatric medications after discharge. Similarly, Maunder [53] documents effects of lockdown for SARS, Pfefferbaum et al. [61] for H1N1, and Jeong et al. [39] for MERS. However, other sets of studies suggest that mental health deterioration follows from policy restrictions instead. Indeed, some evidence establishes that lockdown give rise to feelings of boredom, frustration, and isolation from the rest of the world [9, 10]. Hawryluck et al. [36] found that, during the SARS quarantine in Canada, symptoms of post-traumatic stress disorder and depression were observed in 28.9% and 31.2% of respondents, and longer durations of quarantine were associated with increased prevalence of these symptoms.^4^

Nonetheless, individuals are more prone to comply with government recommendations if they believe that their behaviors have a relevant impact on society [55, 56]. Government recommendations run the risk of exacerbating the fear of contagion and compulsive behaviors [28]. Fear of contagion includes the fear of passing COVID-19 on to other family members [5, 18], which might extend beyond the duration of lockdown [39]. In the UK, during lockdown, 66% of individuals stated that they preferred not to watch the news, because it negatively affected their mental health [69].

### Lockdown due to COVID-19 outbreak

The evidence of mental health effects of COVID-19 reveal wide heterogeneity. Brooks et al. [12] conclude that most of the studies reviewed reported negative psychological effects, such as post-traumatic stress, confusion, and anger. Using Google trends data for Europe and the United States, Brodeur et al. [11] found a substantial increase in search intensity for boredom, loneliness, worry, and sadness, although searches for stress, suicide, and divorce, on the other hand, decreased. Adams-Prassl et al. [2] compared US states that had established strict confinement with those that had not, finding a slight worsening of mental health indicators in the former.

In the UK, Pierce et al. [62] observe that mental distress increased after only one month of lockdown exposure, and Banks and Xu [4, 6] report greater negative effects of lockdown on mental wellbeing among young adults and women, which already exhibited poorer mental health prior to COVID-19. In New Zealand, Sibley et al. [66] explored the immediate effects of a lockdown by comparing samples of New Zealanders assessed before and during the first 18 days of lockdown, finding that people in the pandemic lockdown group reported higher rates of mental distress compared to people in the pre-pandemic group before lockdown.

Nonetheless, such detrimental mental health effects of lockdown are concentrated in some population groups. Codagnone et al. [20] estimate the extent to which the socioeconomic background of a household can predict perceived stress and anxiety using a multi-country (Italy, Spain, and UK) survey, finding that around 42.8% of the population  is at risk of adverse mental health effects due to the combined effect of lockdown and socioeconomic vulnerability. Zhang et al. [71] document that those who stopped working during COVID-19 in China reported worse mental and physical health. Béland et al. [8] confirmed similar results with evidence from Canada, and Gopal et al. [32] and Etheridge and Spantig [23] document an increase in symptoms of anxiety and depression among women in India and UK, respectively. In contrast, Planchuelo-Gómez et al. [63] find that the worsening of individuals mental health during the COVID-19 lockdown, eventually vanished among older people.

Some strand of the literature documents no evidence of a worsening of mental health after a lockdown. Bu et al. [13] document no change in levels of loneliness during the strictest lockdown in the UK. Similarly, Luchetti et al. [50] report no significant changes in the average loneliness across three assessments from January to April. Finally, Foa et al. [26] found that the negative effects associated with the outbreak of the pandemic were concentrated in the period before the lockdown. Once the lockdown went into effect, feelings of sadness, stress, and fear declined and happiness, optimism, and contentment increased. These results are in line with those recorded by ‘Britain's mood’, measured weekly (yougov.co.uk) according to which, between 26 March and 4 April 2020, the percentage of people reporting happiness increased from 26% to 29%, and more significantly, those reporting evidence of decreased stress from 48% to 39%. Fancourt et al. [24] report a reduction in symptoms of anxiety and depression over the first 20 weeks after the introduction of the lockdown in England. The highest levels of depression and anxiety occur in the early stages of the lockdown, but decrease fairly rapidly as individuals adapt to the new circumstances.

It is worth mentioning that lockdowns may have had mental health benefits, such as reduced workplace stress, increased autonomy for telecommuters, or improved work-life balance [34]. The use of digital communication, not only as a tool for information but also for leisure, may have helped to ease the burden of the lock-in itself, compared to previous epidemics where the fear of being disconnected from the world was more dramatic [3].

However, there is wide heterogeneity in the mental health effects of lockdowns. Results are found to be more detrimental among women and lower education and income individuals as well as those with pre-existing mental disorders at the beginning of the lockdown. Recchi et al. [64] found an improvement in self-reported wellbeing in France during lockdown compared to previous years, with the exception of blue-collar workers and residents of the Paris area.

## Data and empirical strategy

### Data

Our data comes from a survey launched online through the website https://COVID19-survey.org/ [25]. The questionnaire was translated into 69 languages. The first call of the online survey was published via social media on 20 March 2020, through the accounts of people connected to traditional media (journalists, TV presenters) along with social media influencers, international and national NGOs, and university networks. In the period between March 20 and April 6, 103,153 questionnaires were collected from 178 countries.^5^ All the information collected in the surveys is  freely available at https://osf.io/3sn2k/.^6^

We focus on data from 22 European countries,^7^ which results in a final sample containing 48,434 observations. We have focused our attention to individuals records of individuals residing in European countries, because at the time of the survey, the pandemic was hitting the European continent harder than the Americas (250,516 confirmed cases in Europe vs. 60,834 in America; 11,986 deaths in Europe vs. 813 in America; WHO, 2021). Moreover, European countries have reasonably similar healthcare systems, at least when compared with the rest of the world.

To control for differences in age, gender, education, and income between respondents and population in each country, we use weights in the descriptive statistics and estimations.^8^

#### Dependent variables

First, we draw on a commonly employed depression Index obtained from eight of the questions of the PHQ-9 (Patient Health Questionnaire) that were included in the survey questionnaire; with the exception of the suicidal idea which was not asked.^9^ The Depression Index is calculated by adding the 8 items and rescaling to values between 0 and 100 (average inter-item covariance: 283.55; alpha Cronbach: 0.8776). Second, we examine evidence from an Anxiety Index computed from the answers to following four questions: "nervous when I think in current circumstances", "worried about my health", "worried about the health of my family", and "stressed about leaving my house". Each item is part of a scale taking values between 0 and 5. The Anxiety Index was calculated by adding the four items and rescaling the total to lie between 0 and 100 (average interitem covariance: 219.80; Cronbach alpha: 0.8421). The depression scale is based on the PHQ-9 (Patient Health Questionnaire) validated by Kroenke et al. [44], with the exception of the exclusion of the item relating to suicidal ideation. The anxiety scale has been validated by Kapoor and Tagat [40].

#### Treatment effects

Policy responses are depicted by two different variables. The first variable is the Oxford COVID-19 Government Response Tracker (denoted in the models as “Stringency Index”). This index takes values from 0 to 100 and summarizes information on several different common policy responses that governments have taken to respond to the pandemic, such as school closures and restrictions in movement. The complete description is reported as a footnote to Fig. 1A of the supplementary material.

The second variable measures the date when lockdown at home became effective (see Table A1 of the supplementary material). We define a binary variable that takes the value 1 if, for the day on which the interviewee answered the survey, and lockdown is in force in their country of residence; and the value 0 otherwise.

Risk exposure is measured from “Our World in data”^10^ for the number of confirmed cases, recovered patients and deaths per 1,000,000 inhabitants for each date and country. Holman et al. [37] have reported an increase of acute stress and depressive symptoms as COVID-19 deaths and infected people increased across the United States [37]. The main limitation of using epidemiological data is that there are differences among countries in terms of legislative provision, recording deaths, and reporting deaths, and the number of confirmed cases reported is also related to testing capacity for COVID-19 [70]. We seek to adjust for some of these differences through fixed effects.

Additionally, we use the Pandemic Severity Index: that is, a binary variable that takes the value 1 if the case fatality rate (ratio between deaths and confirmed cases in percentage) is higher than 2%. The Pandemic Severity Index classifies epidemics into five categories, with category 5 being the highest (Department of Health and Human Services [21]). Given that this category was achieved by the 1918 Spanish flue, the variable ‘Pandemic Category 5’ indicates if COVID-19 has reached the ‘worst-case’ scenario pandemic for each day and country.

### Coarsened exact matching

Coarsened exact matching (CEM) is a matching strategy developed by Iacus et al. [38], which reduces the impact of confoundings on observational causal inference. The strategy consists of simultaneously matching using a set of possible confounders which are "coarsened", reducing the number of possible matching values for a given covariate with the aim of increasing the number of matches achieved.^11^

After applying the CEM method, a weighting variable is obtained to equalize the number of observations within the comparison groups, which takes values between 0 and 1. To check the balance of two comparison groups, the multivariate imbalance measure L1 is used, its size depends on the dataset and the selected covariates, and which takes values between 0 (perfect overall balance) and 1 (maximum imbalance), e.g., a larger value represents a larger imbalance between two groups. When good matching occurs, a substantial reduction in L1 is obtained [33].

In our study, CEM has been used to make the two groups of respondents to the online survey before and after the inception of policy restriction statistically equivalent, based on age, gender, marital status, years of education, income, number of people in the household, and comorbidities.^12^ An additional advantage of the CEM estimator over the standard matching procedure is that it allows us to control for unobserved time-invariant factors. This implies that we assume that the outcome variables of interest of the treated and control units in the absence of any treatment reveal the same growth trajectory, e.g., consistently with the parallel trend assumption of the DiD method.

Table A3 in the supplementary material reports the descriptive statistics for the Anxiety and Depression Indexes, as well as their respective items, cross-classified by implementation of lockdown policies and Pandemic Severity Index of category 5.^13^ As expected, countries that do not exhibit lockdown measures alongside a low mortality rate exhibit the lowest levels of anxiety and depression. In contrast, countries where the pandemic has reached category 5 according to the Pandemic Severity Index, but where no lockdown has been decreed, show the highest levels for sleeping problems (47.33), troubles with concentrating (44.58), nervousness when thinking about current circumstances (75.06), and stress about leaving the house (82.39). Interestingly, countries exposed to a lockdown, but low pandemic mortality show moderately low levels of concern for family’s health (60.93) and stress about leaving the house (76.01).

Finally, the survey provides information on sociodemographic characteristics,^14^ though unfortunately, the survey does not collect information on household composition nor marital status and occupation. Table A4 documents comparable descriptive statistics for sociodemographic variables for the total sample and also for the four regional sub-samples.^15^

### Empirical strategy

Our empirical strategy combines evidence from three different methods, namely, we begin with an event study specification to exploit the effect of exogenous changes in policy measures over depression and anxiety levels. Next, we estimate a difference-in-difference (DiD) strategy where we compare individuals interviewed in countries and on dates that differ in the country-specific policy measures. Finally, we draw on a difference in discontinuity design (RDD) to estimate the effect of change in policy stringency.

#### Event study

We estimate two event study specifications. First, to test the adaptation to lockdown, we use the following model:

1 $${Y}_{ict}=\sum_{j=-7}^{j=7}{\gamma }_{0k}{D}_{kc}{L}_{ct}+{\gamma }_{1}{P}_{ct}+\sum_{j=-7}^{j=7}{\gamma }_{2k}{D}_{kc}{L}_{ct}{P}_{ct}+{\gamma }_{3}{X}_{ict}+{C}_{c}+{T}_{t}+{\epsilon }_{ict},$$

where ${Y}_{ict}$ refers to mental health of the individual *i* living in country *c*, who has answered the online survey on date *t.* Our dependent variable (${Y}_{ict}$) refers to either the PHQ-8 Depression Index (or its 8 items) or the Anxiety Index (or its 4 items).

${L}_{ct}$ is a dummy variable taking the value 1 if a lockdown order has come into force for country *c* and day *t*, and 0 otherwise, and ${D}_{kc}$ are dummy variables for the 7 days before/after the lockdown became effective.^16^

${P}_{ct}$ is a dummy variable taking the value 1 if the pandemic has reached category 5 according to the Pandemic Severity Index (i.e., the case fatality rate, which is the ratio between deaths and confirmed cases, is above 2%) for country *c* and day *t*, and 0 otherwise.

To control for differences in composition, ${X}_{ict}$ refers to sociodemographic characteristics (age, gender, marital status, years of education, number of household members, income, and number of comorbidities). Finally, ${C}_{c}$ and ${T}_{t}$ denote country fixed effects and day fixed effects. Robust standard errors clustered at the day levels are obtained. The eighth day before lockdown came into force is the reference period. The sum of the estimated coefficients ${\gamma }_{0k}+ {\gamma }_{2k}{P}_{ct}$ should be interpreted as the effect of being in the *−j*th day before or after lockdown was effective as compared to 8 days before it.

The second event-study model is used to test the effect of increasing fatality rate, and we specify the following:

2 $${Y}_{ict}=\sum_{j=-7}^{j=7}{\delta }_{0k}{D}_{kc}P+{\delta }_{1}{L}_{ct}+\sum_{j=-7}^{j=7}{\delta }_{2k}{D}_{kc}{L}_{ct}{P}_{ct}+{\delta }_{3}{X}_{ict}+{C}_{c}+{T}_{t}+{\zeta }_{ict},$$

where ${D}_{kc}$ are dummy variables for the 7 days before/after the category 5 pandemic level is reached and the other terms have the same interpretation as in previous model. The eighth day before lockdown came into force is the reference period. The estimated coefficients ${\delta }_{0k}+ {\delta }_{2k}{P}_{ct}$ should be interpreted as the effect of being in the *−j*th day before or after the day in which fatality rate exceeded 2% as compared to eight days before it.

#### Difference-in-difference specification

To disentangle the effect of policy measures on anxiety/depression alongside exposure to a pandemic shock, we rely on a difference-in-difference specification that compares the mental wellbeing of individuals before/after lockdown and before/after the fatality rate reached level 5 in the Pandemic Severity Index. We propose the following DID model:

3 $${Y}_{ict}={\beta }_{0}{L}_{ct}+{\beta }_{1}{P}_{ct}+{\beta }_{2}{L}_{ct}{P}_{ct}+{\beta }_{3}{X}_{ict}+{C}_{c}+{T}_{t}+{\varepsilon }_{ict},$$

where ${Y}_{ict}$ refers to mental health of the individual *i* living in country *c*, who has answered the online survey on date *t*. ${Y}_{ict}$ denotes the PHQ-8 Depression Index (or its 8 items) or the Anxiety Index (or its 4 items), while ${L}_{ct}$ is a dummy variable taking the value 1 if a lockdown order has come into force for country *c* and day *t*, and 0 otherwise. ${P}_{ct}$ is a dummy variable taking the value 1 if the pandemic has reached category 5 according to the Pandemic Severity Index (i.e., the case fatality rate, which is the ratio between deaths and confirmed cases, is above 2%) for country *c* and day *t*, and 0 otherwise. Finally, ${X}_{ict}$ refers to sociodemographic characteristics (age, gender, marital status, years of education, number of household members, income, and number of comorbidities). We also include country fixed effects (${C}_{c}$) and day fixed effects (${T}_{t}$). We obtain robust standard errors clustered at the day level.

#### Canonical estimation

The canonical DiD model presumes the existence of two groups, the treated and the control group, two time periods. When a common trend assumption is satisfied, the two-way fixed-effects estimator is a linear combination of treatment effects across treated units. However, such estimates can be biased when treatment effects change over time within treated units [31]. The presence of treatment effect heterogeneity calls for a series of alternative estimators [14]. However, these estimators may have less statistical power than the pooled estimator, and Marcus and Sant'Anna [52] find that when facing a limited number of groups and time periods (as in our case), it may be reasonable to favor the "weaker" version of the parallel trend assumption.^17^

#### Estimating the lockdown effects

The main challenge in estimating the effect of lockdown is that there is a possibility that individuals may escape from it. However, in most of the countries, the implementation was national-wide and not anticipated, and severe fines^18^ were also imposed on those who failed to comply with lockdown orders.

#### Estimating risk-exposure effects

At the date of the survey, all countries had implemented restrictions on international mobility. However, it is possible that some individuals decided to move within the country to escape from *a higher mortality risk.*^19^ Unfortunately, we do not have information about the region of residence, so we cannot control for this directly. Nor can we identify the effect of asymptomatic individuals or that for those with mild symptoms [57].

#### Regression discontinuity and differences in discontinuity design

The advantage of a regression discontinuity design (RDD) is that by evaluating the level of anxiety and depression around the cut-off date when lockdown came into force (or when the pandemic reached category 5), and comparing these levels for individuals who answered the survey just before and just after, it is possible to identify the causal effect of lockdown (or pandemic category 5) on the outcome variables.

Before the estimation, we must verify two assumptions. First, whether the agents were able to manipulate the running variable (or assignment variable). If the individuals were able to choose with exact precision the moment at which they complete the interview around the cut-off point, there would be a self-selection problem. To test this assumption, we run the McCrary [54] test on the running density function of the variable.

The second assumption refers to the *absence of other policy changes at the same cut-off*. If this assumption is violated, the cross-sectional RD estimator would provide a biased estimate of the average treatment effect, because the multiple confounding policies could not be disentangled from each other [35]. This second assumption is much more difficult to contrast than the first one, since the researcher must look for other policies that have taken place simultaneously. In our case, and as already mentioned when describing the the DiD model, there were no elections during the entire period in which the online data were collected, nor were there any announcements of upcoming elections.

An additional consideration is whether the respondent completed the survey before or after the cut-off point. This rises two potential threads. First,‘optimality effects’, which take place when both the treatment and control groups react to the policy, in our case policy restrictions. Second, the so-called ‘Hawthorne effect’, which takes place when control group individuals modify their behavior once they are followed up. These threats are important if individuals in the control group could anticipate an imminent political change (for example, that a lockdown was going to be announced), in which case their anxiety levels and depression would mimic the reaction of those in the treatment group. Hence, at the cut-off point, both the treatment and control groups would shift, giving rise to a discontinuity in the outcome variables. To solve this problem, RD design is typically combined with the difference-in-difference approach.

We will estimate two different discontinuity designs: (1) to study the immediate effect of the lockdown, while considering the evolution of mortality; (2) to study the effect of the pandemic reaching category 5, while considering whether the country has approved lockdown. The DID-RD proposed for estimating the effect of lockdown is the following:

4 $${Y}_{ict}={\theta }_{0}{f\left({\Upsilon }_{ict}\right)L}_{ct}+{\theta }_{1}{P}_{ct}+{\theta }_{2}f\left({\Upsilon }_{ict}\right){L}_{ct}{P}_{ct}+{\theta }_{3}{X}_{ict}+{C}_{c}+{T}_{t}+{\varrho }_{ict},$$

where ${\Upsilon }_{ict}$ is the distance in days from the day the lockdown becomes effective: positive for the days after the lockdown, and negative for the days before the lockdown. Such a distance is computed for each individual *i* living in country *c* who answered the questionnaire on day *t*. The function $f\left(.\right)$ is a polynomial of the distance in days, that allows for different effects left and right of the discontinuity. Although covariates are not necessary, we include them to reduce the variability in the estimation [47]. The interaction ${\theta }_{2}f\left({\Upsilon }_{ict}\right){L}_{ct}{P}_{ct}$ captures the impact of an increase in mortality to category 5 in an environment where containment has already been ordered.

The DID-RD model proposed to study whether there are structural breaks due to the increase in fatality rate above 2% is as follows:

5 $${Y}_{ict}={\theta }_{0}{g\left({\Psi }_{ict}\right)L}_{ct}+{\theta }_{1}{P}_{ct}+{\theta }_{2}g\left({\Psi }_{ict}\right){L}_{ct}{P}_{ct}+{\theta }_{3}{X}_{ict}+{C}_{c}+{T}_{t}+{\upsilon }_{ict},$$

where ${\Psi }_{ict}$ is the distance in days from the day pandemic reached category 5 according to the Pandemic Severity Index: positive for days after this threshold is reached, negative for the days before it.^20^ This distance is computed for each individual *i* living in country *c* who answered the questionnaire on day *t*. The function $g\left(.\right)$ is a polynomial of the distance in days that allows for different effects on each side of the cut-off. The interaction ${\theta }_{2}g\left({\Psi }_{ict}\right){L}_{ct}{P}_{ct}$ measures the impact of containment in a situation of high mortality.

In estimating (4) and (5), we run a non-parametric local linear kernel regression not assuming any underlying functional form with a triangular kernel, because this method reduces bias in kernel regression methods [47].

Additionally, two fundamental issues must be addressed. The first is the choice of the polynomial applied to the variable running. The second is the choice of the bandwidth. Regarding the first issue, a certain degree of series smoothing eliminates the influence of outliers, but an inappropriate choice of the order of the polynomial may lead to an inadequate approach to the underlying data generating process. To choose the order of the polynomial, we follow the Akaike (AIC) information along with the Lee and Card [46] approach, which is based on the proximity between the estimated polynomial function and the true distribution of the running variable.^21^

## Descriptive statistics

Figure A1 summarizes the distribution of the dependent and explanatory variables throughout the period of analysis (March 20–April 6). This includes epidemiological variables. In the Fig. 1.1, we show the number of confirmed cases per million inhabitants on the left vertical axis, and the number of recoveries and deceased on the right vertical axis.^22^ On average, throughout the entire period, the number of confirmed cases, recovered cases, and deceased per million inhabitants were 386, 32.64, and 12.13, respectively. However, there is a wide dispersion by geographic region (Table A4), with a maximum in Southern Europe (782.96, 86.29, and 62.99, respectively) and a minimum in Eastern Europe (26.67, 1.27, and 0.31, respectively). The effect on depression/anxiety levels by geographic regions will be analyzed later.

Figure 1.2 (in the supplementary Fig. A1) depicts the evolution of the Depression Index, Anxiety Index and Stringency Index for the whole set of countries. As expected, we find an average increase in the Stringency Index from March 23 to April 1. Throughout the entire period, the Anxiety Index is above the Depression Index, and both show a parallel trend at different times (a decrease on March 26 and increase on April 3).^23^ Figures 1.3, 1.4 and 1.5 show the trends in the items that make up the Anxiety Index and the Depression Index for the average of the countries.^24^

Figure A2 displays the average values of epidemiological variables and the average of the Stringency Index by geographic region. The figure reveals that: (i) the number of confirmed cases and recovered patients per 1,000,000 inhabitants peaks by April 2 for Southern countries, decreases afterwards, but ultimately displays an upward trend; (ii) the number of deaths per 1,000,000 inhabitants reveals a different trend between Southern countries and the rest; (iii) the highest levels of Stringency Index correspond to Southern countries, although compared to the levels at the beginning of the interview period, northern countries have experienced a considerable increase in the Stringency Index.

## Results

### Event study

**Fig. 2:**
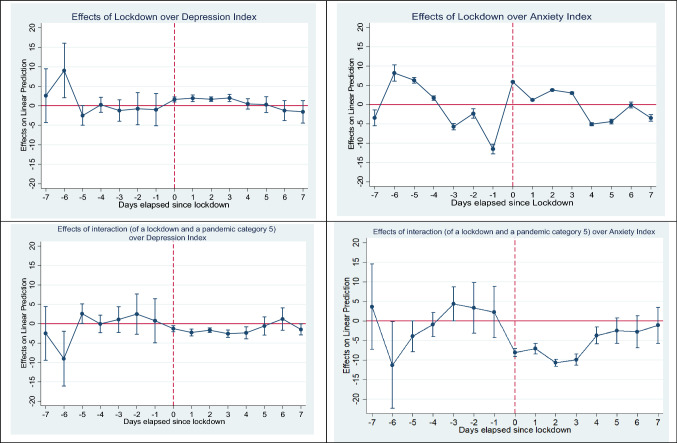
Event study results. Effect of the days before/after lockdown and interaction between days before/after lockdown and pandemic of category 5 over Depression Index and Anxiety Index. The upper panel graphs show the estimated coefficients for $\sum_{j=-7}^{j=7}{\gamma }_{0k}{D}_{kc}{L}_{ct}$ of Eq. 2 for Depression Index (left) and Anxiety Index (right). The lower panel graphs show the estimated coefficients for $\sum_{j=-7}^{j=7}{\gamma }_{2k}{D}_{kc}{L}_{ct}{P}_{ct}$ of Eq. 2 for Depression Index (left) and Anxiety Index (right). See Table 3 for the detail of coefficients and standard deviations. The red dashed line used to signal the day when lockdown became effective

We beging our analysis by reporting an estimate an event study specification including a number of controls,^25^ day fixed effects and country fixed effects, weighted robust standard errors and clustered standard errors at the day level. Figure 2 and Table 1 report the results for considering the event of lockdown.

**Fig. 3:**
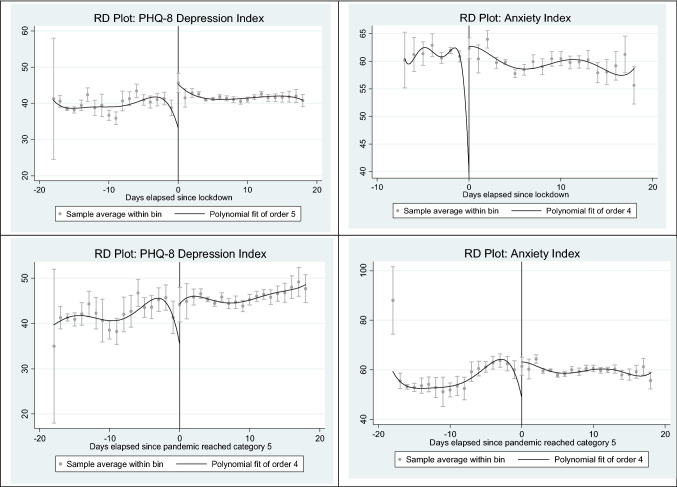
Regression Discontinuity plots for the Depression Index and Anxiety Index. Kernel-weighted local polynomial smoothing discontinuity plot with a triangular kernel. The upper panel graphs show discontinuity for PHQ-8 Depression Index and Anxiety Index around the day when lockdown became into force. The lower panel graphs show discontinuity for PHQ-8 Depression and Anxiety Index around the day when COVID-19 reached category 5 in the Pandemic Severity Index

**Table 1:** Event study results

	Depression index		Anxiety index	
	Effect of days before/after lockdown became effective	Effect of days before/after lockdown became effective and pandemic category 5	Effect of days before/after lockdown became effective	Effect of days before/after lockdown became effective and pandemic category 5
Day − 7	2.616	− 2.481	− 3.408***	3.696
	(3.522)	(3.553)	(1.051)	(5.591)
Day − 6	9.115	− 8.927***	8.260***	− 11.261**
	(3.593)	(3.623)	(1.071)	(5.692)
Day − 5	− 2.486	2.902	6.365***	− 3.899
	(1.272)	(1.292)	(0.380)	(2.014)
Day − 4	0.256	− 0.037	1.711***	− 0.906
	(0.981)	(1.161)	(0.290)	(1.552)
Day − 3	− 1.219	1.072	− 5.673***	4.343
	(1.412)	(1.693)	(0.420)	(2.235)
Day − 2	− 0.734	2.500	− 2.296***	3.329
	(2.094)	(2.657)	(0.630)	(3.321)
Day − 1	− 0.975	0.780	− 11.363***	2.236
	(2.114)	(2.918)	(0.630)	(3.351)
Day lockdown became effective	1.638***	− 1.269***	5.953***	− 8.208***
	(0.330)	(0.410)	(0.100)	(0.590)
Day + 1	1.945***	− 2.260***	1.230***	− 7.000***
	(0.440)	(0.450)	(0.130)	(0.690)
Day + 2	1.701***	− 1.659***	3.803***	− 10.583***
	(0.290)	(0.300)	(0.090)	(0.460)
Day + 3	1.995***	− 2.581***	3.000***	− 9.747***
	(0.470)	(0.480)	(0.140)	(0.741)
Day + 4	0.485	− 2.349	− 5.067***	− 3.708***
	(0.680)	(0.791)	(0.200)	(1.081)
Day + 5	0.298	− 0.567	− 4.374***	− 1.971
	(1.031)	(1.201)	(0.310)	(1.633)
Day + 6	− 1.230	1.209	− 0.102	− 2.595
	(1.312)	(1.462)	(0.390)	(2.064)
Day + 7	− 1.556	− 1.468	− 3.435***	− 4.194***
	(1.472)	(0.731)	(0.440)	(1.121)

Note: Estimated coefficients for days before/after lockdown became effective, and interaction between day before/after lockdown became effective and pandemic of category 5

All models include the following explanatory variables: man, other gender (omitted: women), age and its square, number of years of education and its square, married (omitted: single), specific-country quartile income (omitted: lowest quartile), number of household members (omitted: living alone), number of comorbidities, country fixed effects, and day fixed effects. Individual sample weights have been used to correct for differences in income, education, age, and gender structure between the general population of the country and the corresponding sample. Robust standard errors. ***, **, and * denote statistical significance at the 1%, 5%, and 10% level

On the day the lockdown became effective, we find an increase in the levels of depression and anxiety of 1.63 and 5.95 percentual points (pp), respectively, or an increase by 3.95% and 9.99% with respect to the mean value, respectively. However, the effect of the interaction with pandemic of category 5 is negative for both anxiety and depression. However, the resulting net effect is still positive, although very small for depression (+ 0.89%) and negative for anxiety (-3.78%). These results imply that the immediate effect of lockdown on anxiety symptoms is negative when exposed to a high pandemic mortality.

Figure B1 and Table B1 show the results of an event-study considering the moment in which the pandemic reaches category 5. The immediate effect is an increase in the symptoms of depression (0.893 points) and anxiety (8.220 points), or an increase of 2.16% and 13.80% with respect to the average value, respectively. The effect of the interaction with lockdown exposure is negative in both cases, resulting in a reduction in the symptoms of depression (−1.76% with respect to the mean value) and an increase in the symptoms of anxiety (11.12% with respect to the mean value). Consequently, although increasing mortality rate increase anxiety symptoms beyond those of depression, lockdowns succeed in reducing the increase in anxiety symptoms by almost 20% ((−1.599/8.220)*100).

The reason for this anxiety boost can be found at the core of threat-security theories [30]. Living in an environment with a high mortality risk leads to a perceiving of lockdown in terms of threat-defense that, from a neurophysiological point of view, takes place in the frontal cortex [7]. In this context, complex thinking declines in favor of safety-prioritized decision-making. For this reason, lockdown may no longer be interpreted as a hindrance to individual freedom and the feeling of being safe at home is prioritized.

Our results are in line with those of Michie et al. [55, 56], who found that a better understanding of government recommendations encourages better compliance with them. We go a step further to document that understanding the severity of the situation reduces anxiety levels. More specifically, this takes place by internalizing that staying at home is not an arbitrary imposition (or restriction of individual freedoms) but a protective measure for health.

### Difference-in-difference estimates

Next, we estimate a difference-in-difference model using five different specifications. M1 only includes the binary variables for measuring exposure to a lockdown, exposure to a pandemic of category 5, the interaction between lockdown and pandemic of category 5, day fixed effects, and country fixed effects. M2 includes the same explanatory variables together with gender, age, and its squared term. M3 adds marital status, years of education, and number of household members (omitted: living alone). M4 adds having any comorbidity and number of comorbidities. Finally, M5 includes household income quartile (omitted: lowest quartile)^26^. Estimated coefficients for Depression Index and Anxiety Index are shown in Table 2, and detailed estimations for all the items are reported in Tables C1 and C2.

**Table 2:** Difference-in-difference model

	PHQ-8 depression index					Anxiety Index				
	M1	M2	M3	M4	M5	M1	M2	M3	M4	M5
Lockdown	1.245***	1.235***	1.207***	1.158***	1.242***	4.383***	4.430***	4.401***	4.164***	4.410***
	(0.213)	(0.213)	(0.213)	(0.212)	(0.213)	(0.365)	(0.365)	(0.365)	(0.365)	(0.365)
Pandemic_cat5	2.851***	2.922***	2.911***	2.983***	2.908***	5.916***	5.830***	5.809***	5.872***	5.810***
	(0.217)	(0.217)	(0.217)	(0.216)	(0.217)	(0.371)	(0.372)	(0.372)	(0.372)	(0.372)
Lockdown&Pand_cat5	− 2.582***	− 2.670***	− 2.639***	− 2.608***	− 2.594***	− 5.153***	− 5.069***	− 5.040***	− 5.017***	− 5.018***
	(0.283)	(0.282)	(0.283)	(0.281)	(0.282)	(0.483)	(0.483)	(0.484)	(0.483)	(0.484)
Constant	61.003***	59.226***	59.350***	58.714***	58.630***	64.967***	64.567***	64.115***	63.641***	63.806***
	(0.820)	(0.837)	(0.863)	(0.858)	(0.863)	(1.365)	(1.400)	(1.443)	(1.441)	(1.445)
*N*	44,840	44,840	44,840	44,840	44,840	44,840	44,840	44,840	44,840	44,840
*R*^2^	0.274	0.283	0.283	0.294	0.287	0.213	0.214	0.214	0.217	0.214
*F*	149.047	159.057	143.696	156.373	138.119	23.024	22.064	20.255	23.074	18.901
*p*	0.000	0.000	0.000	0.000	0.000	0.000	0.000	0.000	0.000	0.000

Note: M1 includes lockdown, pandemic of category 5, interaction between lockdown and pandemic of category 5, day fixed effects, and country fixed effects. M2 includes the same explanatory variables than M1, and also, male, other gender (omitted: women), age, and its squared. M3 includes the same explanatory variables than M2 and also married (omitted: single), years of education, and number of household members (omitted: living alone). M4 includes the same explanatory variables than M3 and also having any comorbidity and number of comorbidities. M5 includes the same explanatory variables than M4 and also household income quartile (omitted: lowest quartile). Individual sample weights have been used to correct for differences in income, education, age, and gender structure between the general population of the country and the corresponding sample. Robust standard errors. ***, **, and * denote statistical significance at the 1%, 5%, and 10% level. Lockdown is a binary variable that takes the value one from the day the lockdown becomes effective, and 0 before. Pandemic category 5 is a binary variable if the case fatality rate is higher or equal than 2%. The case fatality rate is the percentage of deceased with respect to confirmed cases. The category 5 corresponds to the highest level of the Pandemic Severity Index. https://www.cdc.gov/media/pdf/mitigationslides.pdf

#### Depression

According to the M5 specification, the Depression Index increases 1.24 pp (2.8% compared to the mean value) after a lockdown has been decreed, and 2.9 pp (7%) once the pandemic has reached level 5 according to the Pandemic Severity Index. However, the joint effect of both situations, that is, lockdown and high mortality, produces a *decrease* in the level of depression by 2.59 pp, which entails a decrease of 6.26% compared to the sample mean.^27^

When we investigate the effect of the different index items, we find that lockdown and pandemic of category 5 mainly increase the incidence of sleeping and concentration problems and also cause alterations in appetite. However, the interaction effect is negative and significant which may indicates that individuals rationalize that lockdown is necessary to overcome the effects of the pandemic, and this internalization process reflects in a decrease in symptoms associated with depression.^28^

#### Anxiety

Although a lockdown increases anxiety level by 4.41 pp (7.4% with respect to the mean value), a more intense effect is observed when the pandemic reaches level 5 (5.8 pp or  9.75% with respect to the mean value). When we look at the items of the Anxiety Index, we find that exposure to a high mortality risk exacerbates nervousness (12.7 pp) and stress from leaving the house (12.234 pp). Lockdown increases individuals concern for family health (4.25 pp) and the stress from leaving home (5.441 pp).

The interaction effect is negative and significant for the Anxiety Index and gives rise to a decrease by 8.43% compared to the sample mean (0.10 standard deviation units). For most of the relevant items, the degree of nervousness decreases by 11.9% with respect to the sample mean (0.13 standard deviation units) and the stress associated with leaving the house decreases by 11.75% (0.12 standard deviation units).

When we examine the effect of the interaction between  Lockdown and Pandemic category 5 for Depression and Anxiety Indexes, we find that it reduces anxiety symptoms levels more intensively (the effect is 1.93 times smaller compared to that of depression symptoms). This "relieving" effect of a lockdown on anxiety has been previously documented, though not in a pandemic setting. Consistently, Eshel et al. [22] found in the context of the Arab–Israeli conflict that the feeling of danger increases feelings of distress, but feeling safe at home decreased the feeling of anxiety.

Interestingly, exposure to a pandemic of category 5 increases concern about family health, but decreases concern for one’s own health. The interaction effect with a lockdown decreases individual′s concern for the health of their family members (−17.26%), but it increases the concern for one’s own health, although the effect is smaller (5.31%). An interpretation of this result is that individuals prioritize the concern for the health of family members over their own. The second result suggests some sort of hypochondriacal behavioral triggered by lockdown.

To further examine the robustness of our findings, we have conducted a test following the spirit of Oster [59], which shows that a positive correlation between the *R*-squared and the absolute size of the coefficients indicates that omitted variables exert a downward bias on the coefficient of interest. Figure B2 shows that as more control variables are included (e.g., more of the variation in the dependent variable is explained by the model), the effect size increases. These results increase our trust in the estimates and, at the same time, justify the use of a comprehensive set of control variables.

#### Heterogeneous effects

Tables 3 and 4 display the results of the difference-in-difference model after conditioning on different sociodemographic characteristics (age, years of education, income, household size, and geographic region^29^) that affect both the Depression and Anxiety Index. For a better understanding of the results, we have computed the effects of the coefficient for lockdown and the interaction in percentage terms with respect to the sample mean and in standard deviation units. The original estimated coefficients are reported in Table C3.

**Table 3:** Effect of lockdown and interaction between lockdown and pandemic of category 5 conditioned on sociodemographic characteristics over PHQ-8 depression index: percentage with respect to sample mean and standard deviation units

	PHQ-8 depression Index		Lockdown coef	Effect of lockdown over depression Index		Interaction coef	Effect of interaction between lockdown and Pan_cat5 over depression Index	
	Mean	In std. dev. units	Table C3	With respect to sample mean (%)	In std. dev. units	Table C3	With respect to sample mean (%)	In std. dev. units
*Age*								
≤ 30 years	47.65	15.86	0.304	0.638	0.012	− 1.444	− 3.030	− 0.068
31–40 years	44.09	14.53	0.322	0.730	0.009	− 1.645	− 3.731	− 0.060
41–50 years	41.48	13.91	1.895	4.568	0.053	− 3.552	− 8.563	− 0.139
51–60 years	39.63	13.36	2.794	7.050	0.102	− 3.818	− 9.634	− 0.187
> 60 years	36.42	12.05	1.884	5.173	0.098	− 2.995	− 8.224	− 0.206
*Education*								
≤ 5 years	43.41	14.76	1.181	2.721	0.068	− 1.013	− 2.334	− 0.090
6–10 years	42.66	14.36	1.424	3.338	0.029	− 2.325	− 5.450	− 0.062
11–15 years	42.80	14.45	2.846	6.650	0.180	0.387	0.904	0.032
16–20 years	43.43	15.27	1.558	3.587	0.045	− 3.283	− 7.559	− 0.134
> 20 years	43.11	14.89	2.885	6.692	0.137	− 4.206	− 9.756	− 0.243
*Income*								
Lowest quartile	46.10	16.19	0.801	1.738	0.023	− 2.436	− 5.284	− 0.091
Second quartile	42.77	14.20	1.769	4.136	0.050	− 2.018	− 4.718	− 0.077
Third quartile	41.71	13.96	1.507	3.613	0.044	− 3.632	− 8.708	− 0.141
Highest quartile	41.04	13.67	0.891	2.171	0.027	− 2.112	− 5.146	− 0.083
*Household size*								
One	45.17	15.51	1.262	2.794	0.042	− 2.967	− 6.569	− 0.132
Two	42.42	14.38	1.103	2.600	0.028	− 2.528	− 5.959	− 0.084
Three	42.81	14.59	1.171	2.735	0.039	− 1.901	− 4.441	− 0.083
More than 3	42.11	14.34	1.302	3.092	0.035	− 2.772	− 6.583	− 0.100
*Region*								
Eastern Europe	43.88	15.10	0.459	1.046	0.004	− 4.112	− 9.371	− 0.329
Northern Europe	42.37	15.06	1.074	2.535	0.031	− 2.932	− 6.920	− 0.245
Southern Europe	44.30	15.08	11.948	26.971	2.464	− 9.688	− 21.869	− 1.425

Note: Estimated coefficients  retrived in the difference-in-difference model (see Table C3) are expressed in terms of percentage with respect to the sample mean and in standard deviation units. The first two columns of the table show the mean and std. dev. of the PHQ-8 Depression Index conditioned on each sociodemographic characteristic. The difference-in-difference model has not been estimated for the sub-sample of Western European countries, because for all countries and dates, lockdown had already become effective

**Table 4:** Effect of lockdown and interaction between lockdown and pandemic of category 5 conditioned on sociodemographic characteristics over Anxiety index: percentage with respect to sample mean and standard deviation units

	Anxiety index		Lockdown coef	Effect of lockdown over anxiety index		Interaction coef	Effect of interaction between lockdown and Pan_cat5 over anxiety index	
	Mean	Std. Dev	Table C3	With respect to sample mean (%)	In std. dev. units	Table C3	With respect to sample mean (%)	In std. dev. units
*Age*								
≤30 years	63.49	24.42	1.725	2.678	0.066	− 4.166	− 6.288	− 0.190
31–40 years	64.04	23.89	1.697	2.695	0.047	− 2.415	− 3.681	− 0.087
41–50 years	62.20	24.20	6.338	10.836	0.191	− 6.449	− 9.699	− 0.237
51–60 years	60.73	24.47	6.229	10.896	0.243	− 5.962	− 9.233	− 0.276
> 60 years	60.86	24.64	6.424	11.234	0.356	− 8.760	− 13.133	− 0.549
*Education*								
≤5 years	62.66	24.44	6.004	10.156	0.386	− 6.748	− 10.043	− 0.575
6–10 years	62.83	24.25	3.685	6.081	0.080	− 5.140	− 7.761	− 0.134
11–15 years	63.11	24.05	3.775	6.208	0.262	− 5.867	− 8.751	− 0.469
16–20 years	61.59	24.31	4.978	8.485	0.156	− 6.445	− 9.791	− 0.255
> 20 years	64.09	24.22	2.716	4.353	0.139	− 3.365	− 5.074	− 0.192
*Income*								
Lowest quartile	63.50	24.50	4.861	8.027	0.149	− 6.179	− 9.129	− 0.224
Second quartile	62.62	24.16	3.944	6.547	0.119	− 4.266	− 6.522	− 0.160
Third quartile	62.63	24.23	3.578	5.917	0.112	− 4.883	− 7.417	− 0.185
Highest quartile	62.00	24.15	4.338	7.300	0.140	− 5.455	− 8.318	− 0.208
*Household size*								
One	62.15	24.54	4.874	8.225	0.172	− 5.083	− 7.763	− 0.218
Two	63.05	24.19	3.210	5.255	0.086	− 4.636	− 7.012	− 0.151
Three	63.10	24.06	3.408	5.585	0.122	− 4.505	− 6.818	− 0.198
More than 3	62.37	24.30	5.642	9.556	0.166	− 6.852	− 10.234	− 0.238
*Region*								
Eastern Europe	62.57	24.86	5.123	8.606	0.263	− 0.342	− 0.545	− 0.001
Northern Europe	61.94	24.62	2.913	4.840	0.090	− 8.338	− 12.339	− 1.148
Southern Europe	65.87	24.67	8.272	13.596	0.833	− 10.996	− 14.858	− 0.947

Note: Estimated coefficients retrived in the difference-indifference model (see Table C3) are expressed in terms of percentage with respect to the sample mean and in standard deviation units. The first two columns of the table show the mean and std. dev. of the Anxiety Index conditioned on each sociodemographic characteristic. The difference-in-difference model has not been estimated for the sub-sample of Western European countries, because for all countries and dates, lockdown had already become effective

#### Age

We find that lockdowns affect mainly the cohorts aged 40 and older. The effects is larger on anxiety: between 4% and 7% for depression, between 10% and 11% for anxiety, compared to the average levels for each cohort. However, the effect of the interaction between lockdown and pandemic mortality level 5 is significant and negative, and in some cases the magnitude larger than that of the lockdown coefficient. For example: depression sypmtoms decrease by 9.634% in the cohort of 51–60 years and anxiety symptoms decreases by 13.133% in the cohort of over 60 years. It should also be noted that for the cohort younger than 30 years, lockdowns cause an increase in anxiety symptoms of 2.67% with respect to its mean level, but the effect of the interaction between lockdown and pandemic mortality level 5 implies a reduction of almost 6.3% (0.17 standard deviation units).

#### Education

Lockdowns exert heterogeneous effects by individuals’ education attainment. For instance, we find that among those in the lowest education attainment, lockdowns increases the Anxiety Index by 10% compared to the average level, while the Depression Index increases by 2.7%. In contrast, among the highest educated, the Depression Index increases by 6.7%, whilst the Anxiety Index increases by 4.4%). The effects of the interaction between lockdown and pandemic mortality level 5 reveal a reduction in the Depression Index among the highest educational group (− 9.7%) and a decrease in Anxiety Index among the lowest educational group (− 10%). Conversely, the lowest reduction in anxiety corresponds to the group with the highesteducation attainment (− 5%).

#### Household income

Next, when we examine the heterogeneous effects of lockdowns by income quartiles, we find evidence of an inverse ∩ -shaped pattern in depression, but a ∪ -shaped pattern in anxiety. In other words, households located at the ends of the distribution show smaller raises in depression levels, but higher raises in anxiety levels. In particular, lockdown increases anxiety by 8% (compared to the mean value) among households in the lowest income quartile, but only increases depression levels by 1.7%. The interaction effect implies a reduction in anxiety levels by 9% (8.3%) for the households with the lowest (highest) income level.

To verify the effect of household income level on levels of anxiety and depression, a difference-in-difference-in-difference model has been estimated by introducing triple interactions between lockdown, category 5 pandemic, and income quartiles. The results are shown in Table C4. Taking the fourth quartile (highest) as a reference, it seems that households with the lowest income reveal the most vulnerable to mental wellbeing deterioration. The effect of lockdown in a category 5 pandemic situation gives rise to an increase of 4.7% (25.8%) in the symptoms of depression (anxiety) among the lowest income households.

#### Household size

Lockdowns give rise to an increase in depressive symptoms by 2% or 3% compared to the average levels. Again, the effect on anxiety is much larger (8.2%) among those living alone and 9.5% among households with more than three members. The effect of the interaction is negative and putweights the coefficient of lockdowns. For example, among people living alone, we estimate a decrease depression symptoms of 6.6% with respect to the average level (that is, more than twice the effect of lockdown). Among households with more than three members, the Anxiety Index decreases by 10% with respect to the mean  which compares to the effect of lockdown.

#### Regional effects

In Western Europe, all countries examined had already implemented a lockdown. Lockdowns considerably increase depression and anxiety in the Southern European  (SE) countries (26.9% and 13.6%, respectively, with respect to the mean value). In comparison, symptoms of depression only increases by 1% in the Eastern European (EE) countries and 4.8% in the Northern European (NE) countries. The effect of the interaction is negative and significant in all regions.

For SE countries, this negative effect almost cancels out the positive effect of lockdowns on depression and it is even higher for anxiety. For the NE countries, the effect of the interaction almost triples (in absolute value) the effect of lockdown on anxiety (− 12.239% compared to 4.840%). For EE countries, the effect of interaction is nine times greater (in absolute value) than the effect of lockdown associated with depression (− 9.4% compared to 1%).

#### Robustness check: the effect of the approval to prescribe chloroquine and hydroxychloroquine to hospitalized patients

As a robustness check, we have studied the joint effect of lockdown and the US Food and Drug Administration (FDA) approval to prescribe chloroquine and hydroxychloroquine to patients hospitalized with COVID-19. This approval took place on 28 March 2020 [48], but was reported in the media on 30 March 2020.^30^ The underlying idea is that the availability of a drug may have affected mental health too. In the case of vaccines, Karayürek et al. [41] and Perez-Arce et al. [60] document that the availability of a vaccine (even before individuals being vaccinated) significantly reduced levels of mental distress. Therefore, we want to make sure that the observed effects on anxiety and depression levels are genuinely caused by lockdown policies. The following difference-in-difference model is estimated:

6 $${Y}_{ict}={\beta }_{0}{L}_{ct}+{\beta }_{1}{HI}_{ct}+{\beta }_{2}{L}_{ct}{HI}_{ct}+{\beta }_{3}{X}_{ict}+{C}_{c}+{T}_{t}+{\varepsilon }_{ict},$$

where ${Y}_{ict}$ refers to mental health of the individual i living in country *c*, who has answered the online survey on date *t*. ${Y}_{ict}$ denotes the Depression Index (or its 8 items) or the Anxiety Index (or its 4 items), while ${L}_{ct}$ is a dummy variable taking the value 1 if a lockdown order has come into force for country *c* and day *t*, and 0 otherwise. ${HI}_{ct}$ is a dummy variable taking the value 1 after approval of chloroquine and hydroxychloroquine (that is, from March 30 onward) for country *c* and day *t*, and 0 otherwise.

The same sociodemographic characteristics $({X}_{ict})$ as in the previous models, country fixed effects (${C}_{c}$), and day fixed effects (${T}_{t}$), are also included. We obtain robust standard errors clustered at the day level.

Tables C5 and C6 show the estimations for the difference-in-difference model for lockdown and clinical approval to prescribe chloroquine and hydroxychloroquine. First, the magnitude and significance of lockdown is similar to that obtained in Table 2 for Depression and Anxiety Indexes (Tables C1 and C2 and for the respective items). Therefore, the variable lockdown is capturing the genuine effect of lockdown on levels of anxiety and depression. Second, the variable hydroxychloroquine is not significant in any regression. Third, the interaction term is not significant neither for the Depression Index nor for any of its items. Finally, the interaction term is significant for the Anxiety Index (−6.5% with respect to the mean) and the items degree of worry about one’s health and family’s health and feeling stressed about leaving the home (−8.3%, −4.6%, and −3.4% with respect to the means).

### Differences in discontinuity estimates

We begin our analysis by exploring the contemporaneous effect of lockdowns or high mortality through a battery of RD plots. These plots show a first-order polynomial of the adjusted variable above and below the cut-off [when the lockdown becomes effective (upper graphs) or when the pandemic reaches category 5 (lower graphs)], which aim to provide suggestive evidence on the possible existence of a discontinuity in the threshold [16]. The main thing to notice from these graphs is the jump or the discontinuity around the cut-off, but no discontinuities are observed before or after (Fig. 3).

As noted earlier, the running variable (days elapsed since lockdown or since pandemic reached category 5) will only be valid if it is not manipulated by individuals. The McCrary density test does not identify any jump in the running variable at the cut-off point (*p* < 0.001) before/after lockdown became effective (upper Fig. D1) and before/after pandemic reached category 5 (lower Fig. D1, which confirms that there are no signs of manipulation (non-random sorting).^31^ Hence, assuming that individuals randomly participate in online surveys, any difference in the outcome variables is due to the effect of the lockdown (or the effect of the pandemic reaching category 5), and therefore, exposure to the treatment is a deterministic function of the calendar day in which they answered the survey.

Another fundamental assumption of an RD design is that baseline covariates should be balanced to preserve the characteristics of the local quasi-natural experiment, that is, all observable and unobservable characteristics of individuals should have a similar distribution around the cut-off as the bandwidth gets narrower [47]. This implies that the imposition of the lockdown or a change in pandemic mortality should not affect the distribution of the covariates around the cut-off. To test for this assumption, an RD model has been estimated in which each covariate acts as a dependent variable (with different bandwidth sizes). Results (available upon request) reject the hypothesis that the baseline covariates are unbalanced around the cut-off point.

Table 5 shows the results of the RD design using a local quadratic regression with a triangular kernel function (Tables D1 and D2 for the items of the Depression Index and Anxiety Index). For each dependent variable, we show the sensitivity to different bandwidths approaches (MSE and CER methods). As mentioned before, the inclusion of baseline covariates can reduce the variability of the estimates, but without affecting the estimation of the jump in discontinuity, regardless of the correlation with the outcome variables [47]. Hence, as a robustness check, we also test the sensitivity of our results due to the inclusion of the baseline covariates and perform two falsification tests using two false thresholds (2 days before and 2 days after the real cut-off points).

**Table 5:** RD design

	MSE optimal	CER optimal	Without covariates	Alternative bandwidth		False threshold	
				6 days	4 days	2 days before	2 days after
*Running variable: days elapsed since lockdown*							
PHQ-depression index							
Lockdown	1.730***	1.741***	1.736***	1.727***	1.719***	1.951	1.635
	(0.341)	(0.331)	(0.321)	(0.351)	(0.341)	(2.050)	(1.533)
Lockdown*Pan_cat5	− 2.165***	− 2.174***	− 2.168***	− 2.155***	− 2.149***	− 0.899	− 1.022
	(0.725)	(0.705)	(0.685)	(0.725)	(0.735)	(1.574)	(1.605)
* N*	19.762	19.762	19.762	22.240	15.242	8.984	22.616
Bandwidth	5	5	5	6	4	5	5
Anxiety index							
Lockdown	3.854***	3.863***	3.849***	3.835***	3.826***	3.739	3.336
	(0.928)	(0.949)	(0.918)	(0.939)	(0.949)	(2.573)	(2.363)
Lockdown*Pan_cat5	− 6.768***	− 6.780***	− 6.769***	− 6.762***	− 6.757***	− 4.868	− 6.128
	(1.657)	(1.677)	(1.636)	(1.615)	(1.595)	(3.708)	(4.670)
*N*	19.762	19.762	19.762	22.240	15.242	8.984	22.616
Bandwidth	5	5	5	6	4	5	5
*Running variable: days elapsed since pandemic reached category 5*							
PHQ-depression index							
Pan_cat5	2.352***	2.356***	2.342***	2.305***	2.303***	2.279	2.369
	(0.654)	(0.674)	(0.685)	(0.705)	(0.715)	(1.533)	(1.523)
Lockdown*Pan_cat5	− 1.847***	− 1.855***	− 1.841***	− 1.826***	− 1.823***	− 1.227	− 1.470
	(0.573)	(0.583)	(0.593)	(0.614)	(0.624)	(2.426)	(2.165)
Obs. Left	19.762	19.762	19.762	22.240	15.242	8.984	22.616
Bandwidth	5	5	5	6	4	5	5
Anxiety index							
Pan_cat5	8.487***	8.492***	8.475***	8.458***	8.451***	6.298	5.450
	(0.949)	(0.939)	(0.959)	(0.979)	(1.000)	(4.659)	(3.934)
Lockdown*Pan_cat5	− 2.205***	− 2.209***	− 2.198***	− 2.173***	− 2.166***	− 2.140	1.699
	(0.492)	(0.503)	(0.503)	(0.482)	(0.553)	(1.564)	(1.225)
*N*	19.762	19.762	19.762	22.240	15.242	8.984	22.616
Bandwidth	5	5	5	6	4	5	5

Note: Mean square error (MSE): optimal bandwidth is estimated by taking the minimum optimal bandwidth of the most common MSE-optimal procedures. Coverage error (CER): optimal bandwidth is the minimum bandwidth of the different coverage error procedures following Calonico et al. [17]. Robust standard errors. ***, **, and * denote statistical significance at the 1%, 5%, and 10% level, respectively

Using the results from the means square method (MSE) estimation method, we document that lockdowns give rise to an increase in depression and anxiety symptoms (1.7pp and 3.8pp, respectively; or *4.2% and 6.5% with respect to the mean value*). Consistently with previous results, the effect of the interaction is negative, resulting in a reduction in the level of depression (−5.22% with respect to the mean value) and, to a much larger extent, a reduction in anxiety symptoms (−11.4%).

Comparing the estimates of the interaction term (Lockdown*Pan_cat5) we find that for both the Depression and the Anxiety Index, the effect (in absolute value) is larger when the running variable is "days elapsed since lockdown" compared to "days elapsed, since pandemic reached category 5" (−2.2pp vs. −1.8pp for depression; −6.7 vs. −2.2 for anxiety) which suggests that individuals internalize the need of a lockdown when they perceive a higher threat of the pandemic.

Results from the converage error (CER) method mirror those of the MSE method. A high mortality environment leads to an increase in the symptoms of depression and anxiety (2.4pp and 8.5pp, respectively). Although lockdowns exert a certain mitigating effect on these increases, the resulting net effect is an increase in the symptoms of depression (1.2% with respect to the mean value) and anxiety (10.5% with respect to the mean value). Therefore, the results of RD design are consistent with those obtained in event studies.^32^

Comparing the results with and without baseline covariates (including only fixed effects), renders no appreciable differences. These effects are robust across different bandwidth sizes, near the cut-off point. Importantly, we do not obtain significant results when using alternative false cut-offs.

When we turn to the specific items of the Depression Index (Table D1), we observe that lockdown increases problems relating to sleeping and concentrating + 6.6% and 7.6% 

with respect to mean values, respectively). However, if lockdowns coexists with a high pandemic mortality risk, the resulting net effect turns negative (−5% and −2.7% with respect to mean values, respectively).

Yet, when the pandemic reaches level 5, we find an increase in the probability of feeling down (7.2% increase with respect to the mean value), sleep problems (12%), appetite (7.4%), and concentration (13.8%). However, unlike in the previous model, if a lockdown is simultaneously decreed with a pandemic level, it does not give rise to a marked reduction in depressive symptoms.^33^

Table D2 shows the results of the RD design for each item of the Anxiety Index. Living in a lockdown fundamentally increases individuals concerns for the health of the family and, the stress associated with leaving home (given the fear of contagion). However, when it coincides with a level 5 pandemic, the effect on concern for the family fades, and the stress from having to leave home is greatly atenuated. Therefore, lockdown measures are interpreted as a health protective measure.

### Limitations

We are aware that this study has some limitations. First, we rely on self-reported data by survey participants. It has not been possible to ascertain whether any medical diagnosis was made to participants after the lockdown, nor how pre-existent subclinical symptomatology in the weeks or months prior to the lockdown affected the responses collected in the survey. Second, the survey is not designed to elicit data on health during the pandemic, e.g., the precise scale values cannot be taken as national averages of anxiety and depression during the pandemic. As the data collection was conducted through an online survey, participants who did not have access to the Internet at home were not represented. Therefore, if there is selection on fixed unobservables that systematically differ between internet users and non-users (e.g., that individuals who were more worried about the COVID-19 pandemic were disproportionately more likely to take or share this survey), then our estimates will be biased by the unobservable components of changes in mental health among internet users. To address this problem, observations have been weighted to improve their representativeness at the country level. Weights vary according to the respondents' gender, age, income, and education. Additionally, to check the robustness of our findings, we have performed a test following the spirit of Oster [59], which shows that a positive correlation between the *R*-squared and the absolute size of the coefficients indicates that omitted variables exert a downward bias on the coefficient of interest. Figure B2 show that as more control variables are included (e.g., more of the variation in the dependent variable is explained), the effect size increases. These results increase confidence in our estimates and, at the same time, justify the use of a comprehensive set of control variables.

## Conclusions

Using self reported mental wellbeing data from March to April 2020 that identifies the effect of exposure to COVID-19 and lockdown stringency across a number of European countries, we have examined the so-called ‘welcomed lockdown hypothesis’. That is, we have tested  the extent to which there is a specific level of risk exposure where the effect of mobility restrictions (lockdown level 5) improves, or at least does not deteriorate mental health. We have drawn on three specifications, namely an event study, a difference-in-difference (DiD) and differences in discontinuity designs to identify the effects. From a methodological perspective, our analysis highlights some interesting properties of the CGE, which should make it worthy of consideration when assessing the effectiveness of public policies using quasi-experimental data (i.e., online surveys).

Our findings show that while a ‘preventive’ lockdown in a low/moderate mortality environment increases in symptoms of depression and anxiety. However,  in a high-mortality setting (such as those in many countries during the first wave), lockdowns mitigate such negative effects, particularly detrimental effects on anxiety.

All efforts to overcome interpersonal isolation play an important role at times of high stress and strain [27]. There is evidence that having a telephone support line, staffed by psychiatric nurses, set up specifically for people in quarantine could be effective in providing them with a social network. For example, in both China and Korea, mental health professionals quickly and widely established online counselling services to provide free 24/7 services and online self-help intervention systems, including cognitive behavioral therapy for depression, anxiety, and insomnia [45, 49].

The use of the media also plays an important role in disseminating information about the pandemic [29], hence helping people to understand the need of a lockdown. Health policymakers should pay more attention to the effects of mobility restrictions on depression and anxiety among the general population, and specifically address the problem of inadequate information or “infodemia” during public health emergencies.

Another strategy to minimize the negative effects of lockdowns on mental health includes the design of optimal differential policies along the lines of those recommended by Acemoglu et al. [1], taking into consideration not only the rate of infection, hospitalization and fatality rate for different population groups, but also differentiating between groups with higher or lower exposure to mental health risks.

## Supplementary Information

Below is the link to the electronic supplementary material.

Supplementary file1 (DOCX 330 KB)

## Footnotes

1 Millions of people could die if the coronavirus pandemic sees a second wave of infections. “The a, which behaved exactly like COVID-19: it went down in the summer and fiercely resumed in September and October, creating 50 million deceased during the second wave”. Available at: https://edition.cnn.com/world/live-news/coronavirus-pandemic-06-26-20-intl/h_337aac61bfd759992dd23cd77cbd00ed.

2 Red figures depict mortality per million inhabitants, green circles denote the spread of the virus (confirmed cases per million inhabitants), and the heights of the blue bricks indicate the value of the COVID-19 Government Response Stringency Index (abbreviated “Stringency Index”).

3 Our choice to use CEM is motivated by the fact that this method has been designed by the authors to provide an improvement over existing matching approaches in estimating causal inference by reducing any imbalance in covariates between treated and control units. CEM incorporates properties of the exact matching procedure, and in addition, it possesses a peculiar feature that distinguishes it from other matching methods in allowing the balance between treated and control groups to be chosen ex-ante.

4 Such effects result in part from difficulties in obtaining supplies, problems in receiving medical treatment, or for other reasons not related to the health emergency [9].

5 Pierce et al. [62] also use data from an only survey but with a shorter interview window (April 23rd-30th).

6 We thank Fetzer et al. [25] for the availability of the database and the description of the questionnaire.

7 Austria, Belgium, Bulgaria, Czech Republic, Denmark, Finland, France, Germany, Greece, Hungary, Ireland, Italy, Netherlands, Norway, Portugal, Romania, Slovakia, Spain, Sweden, Switzerland, Ukraine, and United Kingdom.

8 For each individual I, a weight is assigned according to the category to which he or she belongs (age–sex–income–education–country)

$${\mathrm{weight}\_i}_{\mathrm{age},\mathrm{ gender},\mathrm{income},\mathrm{ educ},\mathrm{country}}=\frac{{F}_{\mathrm{age},\mathrm{gender},\mathrm{income},\mathrm{educ},\mathrm{country}}\bullet {N}_{\mathrm{country}}}{{N}_{\mathrm{age},\mathrm{ gender},\mathrm{ income},\mathrm{ educ}}};$$

$${F}_{\mathrm{a}\mathrm{g}\mathrm{e},\mathrm{g}\mathrm{e}\mathrm{n}\mathrm{d}\mathrm{e}\mathrm{r},\mathrm{i}\mathrm{n}\mathrm{c}\mathrm{o}\mathrm{m}\mathrm{e},\mathrm{e}\mathrm{d}\mathrm{u}\mathrm{c},\mathrm{c}\mathrm{o}\mathrm{u}\mathrm{n}\mathrm{t}\mathrm{r}\mathrm{y}}$$

is the fraction of the population for each category age–gender–income–education for each country [considering 11 categories were considered for age (18–19, 20–24, 25–29, 30–34, 35–39, 40–44, 45–49, 50–54, 55–59, 60–64, 65 +), country-level income quintiles and three categories for education (less than 8 years of education, between 9 and 14 years of education, more than 14 years of education)]; *N*_country_is the number of observations in the country; *N*_age, gender, income, educ_, is the number of individuals in the survey for each combination age–gender–income–education in each country. These weights give a higher weighting to categories where there are fewer observations in the survey and to categories corresponding to a larger fraction of the country's population.

9 Information is available for the remaining eight items: "little interest or pleasure in doing things", "feeling down or hopeless", "trouble falling asleep or staying asleep or sleeping too much", "feeling tired or having little energy", " poor appetite or overeating”, feeling bad about oneself (or that you are a failure or have let yourself or your family down)”,“trouble concentrating on things, such as reading the newspaper or watching television”, and “moving or speaking so slowly that other people could have noticed or so fidgety or restless that you have been moving a lot more than usual”.

10 https://ourworldindata.org/coronavirus-data-explorer.

11 CEM works as follows. First, it makes a copy of the set of covariates chosen for matching. Second, the variables are broken down into different meaningful strata (i.e., into equal intervals of the same size or into intervals of different dimension from each other), through user choice automatically or through the CEM algorithm. Third, a unique stratum is created for each observation and each observation is placed in a stratum. The strata created are reassigned to the original data set, and any strata that do not contain at least one treated and one control unit are removed. Thus, the treatment effect is based on the matching provided by the algorithm, since the difference between treated and control units is obtained from the difference in the outcome variable between units belonging to the same strata. Finally, the higher the coarsening (higher number of strata), the lower the imbalance, as well as the lower the number of matches provided by the CEM.

12 Muennig et al. [58] and Tetteh et al. [68] have found that CEM is preferable to other matching procedures (e.g., propensity score matching) in terms of more efficient processing and reduced model dependence, variance, and bias. Ripollone et al. [65] also showed that optimal performance is warranted only when the vector of important confounders is relatively small (fewer than 10), which is fulfilled in our case.

13 Table A3 also compares the Depression Index and Anxiety Index between the initial sample and the sample obtained after applying CEM.

14 These includes age, gender, marital status, number of years of education, number of household members, number of comorbidities (cardiovascular diseases, diabetes, hepatitis B, chronic obstructive pulmonary disease, chronic kidney diseases, and cancer), and monthly household income before taxes.

15 Table A4 also compares the descriptive statistics between the initial sample and the sample obtained after applying CEM.

16 See Table A1 for the day when lockdown became effective.

17 As the weights are proportional to the residuals from a regression of treatment on country and day fixed effects, we have checked that the residuals from a regression of the outcome variable on region and day fixed effects are linearly related to the residuals from a regression of treatment on region and day fixed effects and the slope of this linear relationship does not differ between the treatment group and the comparison group (results available upon request).

18 Fines jumped to €3000 in Italy, March 25. Retrieved from: https://www.express.co.uk/news/world/1259781/coronavirus-latest-italy-fines-Giuseppe-Conte-boris-johnson-uk-lockdown-COVID-19. Man jailed for violating lockdown rules in France, April 1. Retrieved from: https://www.rfi.fr/en/france/20200401-france-man-jailed-for-violating-lockdown-rules-easter-holidays-cancelled.

19 In the UK, where no lockdown was decreed, people were urged not to move house. https://www.bbc.com/news/business-52051174.

20 See Fig: A3: cells in red and green correspond to days with fatality rate is above 2%.

21 Although in theory a RD model only requires a very small window of observations, an excessively small number of observations can lead to unbiased, but inefficient estimates. Increasing the size of the window increases the estimation efficiency. Therefore, there is a trade-off between the efficiency and the unbiasedness of the estimates [51]. As for the choice of the optimal bandwidth, we have relied on two methods: mean square error (MSE) optimal bandwidth which it is estimated by taking the minimum optimal bandwidth of the most common MSE-optimal procedures [15] and the coverage error rate (CER) optimal bandwidth which is the minimum bandwidth of the different coverage error procedures [17].

22 There is a noticeable increase in the number of confirmed cases per million inhabitants (from 290.35 to 1,123.45), and similarly, a progressive increase in recoveries (from 15.63 to 290.83). For both variables, a maximum is found on March 5. Deceased show a decreasing trend between March 20 and 29, but an increasing trend afterward until reaching 29.25 per million by the end of the period.

23 There is also wide variability between regions (see Table A4). For example, the average Stringency Index is 45.66 in Northern Europe versus 84.28 in Eastern Europe, the Depression Index ranges from 45.03 (Bulgaria) to 37.93 (Finland), and the Anxiety Index ranges from 65.92 (Portugal) to 53.52 (Sweden).

24 Among the anxiety items, the one corresponding to “stressed about leaving one's own house” (mean: 78.19) is above all the others, and among the depression items, those corresponding to “sleeping problems” (mean: 45.70) and “feeling tired” (mean: 48.60) show slightly higher values. At the opposite extreme we find "trouble for concentrating" (mean: 30.10). Table A5 shows the mean of each item by country and geographic region.

25 Controls include male, other gender, age and its squared, married, years of education and number of household members, having any comorbidity and number of comorbidities, household income quartile.

26 Individual sample weights have been used to correct for differences in income, education, age, and gender structure between the general population of the country and the corresponding sample.

27 See Table A2 for mean and standard deviations of the Depression Index, Anxiety Index and their items.

28 For example: sleeping problems decrease by 12.11% with respect to the sample mean; concentration problems decrease by 10.88%, appetite disorders decrease by 6.22%.

29 The difference-in-difference model has not been estimated for the sub-sample of Western European countries because for all countries and dates, lockdown had already become effective.

30 Coronavirus (COVID-19) Update: Daily Roundup March 30, 2020 | FDA; FDA authorizes emergency use of unapproved drugs to treat coronavirus—The Washington Post; March 30, 2020 coronavirus news (cnn.com); who-audio-emergencies-coronavirus-press-conference-full-30mar2020.pdf.

31 The McCrary’s test consists in estimating the density function on either side of the cut-off point. Observing a discontinuous density at the cut-off provides evidence of manipulation. After computing the discontinuity at the cut-off point and its standard error, a *t*-test of no statistical significance is constructed, where the null hypotheses is no statistical evidence of discontinuity in the density function at the cut-off point. In our sample, we estimate the McCrary’s test and we define as no manipulated survey respondents those individuals where there is no statistical evidence of discontinuity in the density function at the cut-off (i.e., McCrary’s test of the null hypothesis fails to be rejected). McCrary test validates the empirical strategy (the test statistic is 0.991/0.740 for the upper figures and 0.867/0.619 for the lower figures).

32 It should be noted that a direct comparison cannot be made between the DID and RD design estimates. In the first case, all pre-lockdown observations (pre-pandemic of category 5) are compared with all post-lockdown observations (post-pandemic of category 5). In the second case, RD design captures the immediate effect in a few days around the cut-off point.

33 For example, the interaction is not significant for sleep problems and the net effect continues to be positive for feeling down (3.76%), appetite problems (2.62%) and concentration problems (5.13%).
