# Supplementary material for: The ‘welcomed lockdown’ hypothesis? Mental wellbeing and mobility restrictions
Source: Eur J Health Econ. 2022 Aug 12;24(5):679–99. doi: 10.1007/s10198-022-01490-6 (PMC9371965; doi:10.1007/s10198-022-01490-6)
Supplement: Supplementary file 1 — Supplementary file1 (DOCX 330 KB) [file 10198_2022_1490_MOESM1_ESM.docx]

**Appendix A**

**Figure A1. Epidemiological variables of COVID-19, PHQ-8 Depression Index, Anxiety Index and Stringency Index between March 20^th^ and April 6^th^.**

|  |  |
| --- | --- |
|  |  |
|  |  |

Figure 1.1 represents the number of confirmed COVID-19 cases, recovered cases and deceased per 1,000,000 inhabitants.

Figure 1.2 represents the PHQ-8 Depression Index, Anxiety Index and Stringency Index (COVID-19 Government Response Stringency Index). Source: [https://COVID19-survey.org/results.html](https://covid19-survey.org/results.html)for Depression Index (PHQ-8) and Anxiety Index; [https://www.bsg.ox.ac.uk/research/research-projects/oxford-COVID-19-government-response-tracker](https://www.bsg.ox.ac.uk/research/research-projects/oxford-covid-19-government-response-tracker) for Stringency Index.

Figure 1.3 represents the four items of the Anxiety Index.

Figure 1.4 represents items 1 to 4 of the PHQ-8 Depression Index.

Figure 1.5 represents items 5 to 8 of the PHQ-8 Depression Index.

Individual sample weights have been used to correct for differences in income, education, age and gender structure between the general population of the country and the corresponding sample.

The Stringency Index is a composite measure obtained by additive score of nine indicators measured on an ordinal scale, and rescaled afterwards in order to vary from 0 to 100. The nine items included are the following ones:

1. School closing (0: no measures; 1: recommend closing; 2: require closing); Workplace closures (0: no measures; 1: recommend closing; 2: require closing for some sectors or categories of workers; 3: require closing all but essential workplaces).
2. Cancel public events (0: no measures; 1: recommend cancelling; 2: require cancelling); restrictions on gatherings (0: no restrictions; 1: restrictions on gatherings above 1,000 people, 2: restrictions on gatherings between 100 and 1,000 people; 3: restrictions on gatherings between 10 and 100 people; 4: restrictions on gatherings of less than 10 people).
3. Close public transport (0: no measures; 1: recommend closing or significantly reduce volume or transport available; 2: require closing or prohibit most citizens from using it).
4. Public information campaigns (0: no public information campaign; 1: public officials urging caution about COVID-19; 2: coordinated public information campaign across traditional and social media).
5. Stay at home (0: no measures; 1: recommend not leaving house; 2: require not leaving house with exceptions for daily exercise, grocery shopping and essential trips; 3: require not leaving house with minimal exceptions).
6. Restrictions on internal movement (0: no measures: 1: recommend movement restriction; 2: restrict movement).
7. International travel controls (0: no measures; 1: screening; 2: quarantine arrivals from high-risk regions; 3: ban on high-risk regions; 4: total border closure).
8. Testing policy (0: no testing policy; 1: only those who have symptoms and meet specific criteria, such as, key workers, admitted to hospital, came into contact with a known case or returned from overseas; 2: testing anyone showing COVID-19 symptoms; 3: open public testing).
9. Contact tracing (0: no contact tracing; 1: limited contact tracing, that is not done for all cases; 2: comprehensive contact tracing, that is, done for all cases).

Depression Index (PHQ-8) is obtained as the sum of 8 items (little interest or pleasure in doing things, feeling down or hopeless, trouble falling asleep or sleeping too much, feeling tired or with little energy, poor appetite or overeating, feeling bad about oneself, trouble concentrating on things such as reading the newspaper or watching television, moving or speaking slowly or fidgety), each of them taking values between 0 and 100. The final sum is also re-scaled to take values between 0 and 100.

Anxiety Index is obtained as the sum of 4 items (nervous when thinking in current circumstances; worried about one’s health; worried about the health of family members; stressed about leaving one’s house), each of them taking values between 0 and 100. The final sum is also re-scaled to take values between 0 and 100.

**Figure A2. Epidemiological variables of COVID-19 and Stringency Index by regions between March 20^th^ and April 6^th^.**

|  |  |
| --- | --- |
|  |  |

Source: Own work using data from: (i) Coronavirus Pandemic Data Explorer <https://ourworldindata.org/coronavirus-data-explorer> for confirmed cases, recovered cases and deceased per 1,000,000 inhabitants (ii) [https://www.bsg.ox.ac.uk/research/research-projects/oxford-COVID-19-government-response-tracker](https://www.bsg.ox.ac.uk/research/research-projects/oxford-covid-19-government-response-tracker) for COVID-19 Government Response Stringency Index (Stringency Index).

Eastern Europe: Bulgaria, Czech Republic, Hungary, Romania, Slovakia and Ukraine. Northern Europe: Denmark, Finland, Ireland, Norway, Sweden and United Kingdom. Southern Europe: Greece, Italy, Portugal and Spain. Western Europe: Austria, Belgium, France, Germany, Netherlands and Switzerland.

**Figure A3. Combination of lockdown measures and fatality rate by date and country**

|  | March | | | | | | | | | | | | April | | | | | |
| --- | --- | --- | --- | --- | --- | --- | --- | --- | --- | --- | --- | --- | --- | --- | --- | --- | --- | --- |
|  | 20 | 21 | 22 | 23 | 24 | 25 | 26 | 27 | 28 | 29 | 30 | 31 | 1 | 2 | 3 | 4 | 5 | 6 |
| Austria |  |  |  |  |  |  |  |  |  |  |  |  |  |  |  |  |  |  |
| Belgium |  |  |  |  |  |  |  |  |  |  |  |  |  |  |  |  |  |  |
| Bulgaria |  |  |  |  |  |  |  |  |  |  |  |  |  |  |  |  |  |  |
| Czech Republic |  |  |  |  |  |  |  |  |  |  |  |  |  |  |  |  |  |  |
| Denmark |  |  |  |  |  |  |  |  |  |  |  |  |  |  |  |  |  |  |
| Finland |  |  |  |  |  |  |  |  |  |  |  |  |  |  |  |  |  |  |
| France |  |  |  |  |  |  |  |  |  |  |  |  |  |  |  |  |  |  |
| Germany |  |  |  |  |  |  |  |  |  |  |  |  |  |  |  |  |  |  |
| Greece |  |  |  |  |  |  |  |  |  |  |  |  |  |  |  |  |  |  |
| Hungary |  |  |  |  |  |  |  |  |  |  |  |  |  |  |  |  |  |  |
| Ireland |  |  |  |  |  |  |  |  |  |  |  |  |  |  |  |  |  |  |
| Italy |  |  |  |  |  |  |  |  |  |  |  |  |  |  |  |  |  |  |
| Netherlands |  |  |  |  |  |  |  |  |  |  |  |  |  |  |  |  |  |  |
| Norway |  |  |  |  |  |  |  |  |  |  |  |  |  |  |  |  |  |  |
| Portugal |  |  |  |  |  |  |  |  |  |  |  |  |  |  |  |  |  |  |
| Romania |  |  |  |  |  |  |  |  |  |  |  |  |  |  |  |  |  |  |
| Slovakia |  |  |  |  |  |  |  |  |  |  |  |  |  |  |  |  |  |  |
| Spain |  |  |  |  |  |  |  |  |  |  |  |  |  |  |  |  |  |  |
| Sweden |  |  |  |  |  |  |  |  |  |  |  |  |  |  |  |  |  |  |
| Switzerland |  |  |  |  |  |  |  |  |  |  |  |  |  |  |  |  |  |  |
| Ukraine |  |  |  |  |  |  |  |  |  |  |  |  |  |  |  |  |  |  |
| United Kingdom |  |  |  |  |  |  |  |  |  |  |  |  |  |  |  |  |  |  |

| White | No lockdown and fatality rate lower than 2%. |
| --- | --- |
| Yellow | Lockdown has become effective and fatality rate lower than 2% |
| Green | No lockdown, but fatality rate higher or equal than 2%. |
| Red | Lockdown has become effective and fatality rate higher or equal than 2% |

Information from lockdown dates obtained from [https://auravision.ai/COVID19-lockdown-tracker/](https://auravision.ai/covid19-lockdown-tracker/). The case fatality rate is the percentage of deceased with respect to confirmed cases. The category 5 corresponds to the highest level of the Pandemic Severity Index. Information of confirmed cases and deceased per 1,000,000 inhabitants obtained from <https://ourworldindata.org/coronavirus-data-explorer>

**Table A1. Dates lockdown became effective**

| **Country** | **Day lockdown became effective** | **Stringency Index before lockdown** | **Stringency Index after lockdown** |
| --- | --- | --- | --- |
| Austria | March 16th | 57.27 | 84.79 |
| Belgium | March 18th | 53.04 | 75.26 |
| Bulgaria | March 13th | 29.49 | 71.83 |
| Czech Republic | March 16th | 48.02 | 67.59 |
| Denmark | March 13th | 79.49 | 84.12 |
| Finland | March 16th | 47.48 | 71.55 |
| France | March 17th | 53.17 | 89.41 |
| Germany | March 17th | 46.30 | 71.83 |
| Greece | March 23th | 57.27 | 76.32 |
| Hungary | March 28th | 59.78 | 78.44 |
| Ireland | March 27th | 57.40 | 79.63 |
| Italy | March 9th | 64.44 | 83.46 |
| Netherlands | March 16th | 48.80 | 74.86 |
| Norway | March 12th | 16.93 | 72.48 |
| Portugal | March 19th | 44.31 | 62.57 |
| Romania | March 25th | 67.06 | 80.95 |
| Slovakia | March 16th | 56.48 | 82.14 |
| Spain | March 14th | 47.10 | 71.69 |
| Sweden | No lockdown | Stringency Index varies between 32.4 and 45.36 | |
| Switzerland | March 17th | 27.38 | 79.49 |
| Ukraine | March 17th | 51.59 | 92.06 |
| United Kingdom | March 24th | 37.83 | 75.13 |

Source: Own work using [https://ourworldindata.org/grapher/COVID-stringency-index](https://ourworldindata.org/grapher/covid-stringency-index) (for Stringency Index) and [https://auravision.ai/COVID19-lockdown-tracker/](https://auravision.ai/covid19-lockdown-tracker/) (for lockdown dates).

**Table. A2. L1 statistic before and after CEM (coarsened exact matching method)**

|  | Initial sample | Sample after CEM |
| --- | --- | --- |
| Man | 0.110(− 0.142) | 1.8e-14 (2.2e-14) |
| Age | 0.022(0.022) | 2.4e-14 (2.4e-14) |
| Years of education | 0.224(0.248)) | 2.4e-14 (− 6.8e-14) |
| Married | 0.049(−0.049) | 4.4e-16 (2.2e-16) |
| Single | 0.097(0.098) | 1.2e-14(2.0e-14) |
| Household size | 0.224(−0.248) | 9.2e-16(1.4e-14) |
| Number of comorbidities | 0.080(0.012) | 4.2e-16(4.4e-14) |
| Income quartile: 1st | 0.049(0.056) | 1.7e-14(−2.9e-14) |
| Income quartile: 2nd | 0.022(−0.022) | 9.4e-14(9.9e-14) |
| Income quartile: 3rd | 0.107(0.112) | 6.7e-16(8.0e-14) |
| Income quartile: 4th | 0.185(0.190) | 7.4e-16(7.8e-14) |
| Multivariate L_1_ | 0.781 | 6.404e-16 |
| N | 48,434 | 44,840 |
| Matched | - | 44,840 (91.54%) |
| Unmatched | - | 4,097 (8.46%) |

Difference in means between parenthesis.

**Table A3. Descriptive statistics for PHQ-8 Depression Index and Anxiety index conditioned on lockdown and Pandemic Severity Index**

|  | All sample | | Lockdown=0 | Lockdown=1 | Lockdown=0 | Lockdown=1 |
| --- | --- | --- | --- | --- | --- | --- |
|  | Mean | Std. Dev. | Pandemic Category 5=0 | Pandemic Category 5=0 | Pandemic Category 5=1 | Pandemic Category 5=1 |
| **Inicial sample** |  |  |  |  |  |  |
| Depression Index | 41.25 | 14.63 | 39.35 | 41.19 | 41.80 | 41.82 |
| 1. Little interest or pleasure in doing things | 43.53 | 21.87 | 43.23 | 44.68 | 42.30 | 43.29 |
| 1. Feeling down | 42.38 | 20.68 | 40.91 | 41.42 | 43.73 | 43.23 |
| 1. Trouble falling asleep or sleeping too much | 45.49 | 23.39 | 42.27 | 45.41 | 47.11 | 45.58 |
| 1. Feeling tired or having little energy | 48.37 | 22.06 | 48.18 | 48.81 | 48.58 | 47.45 |
| 1. Poor appetite or overeating | 41.15 | 22.32 | 38.95 | 41.11 | 41.27 | 42.38 |
| 1. Feeling bad about oneself | 36.20 | 19.64 | 34.25 | 35.44 | 37.07 | 37.60 |
| 1. Trouble concentrating on things | 42.78 | 22.67 | 38.44 | 42.28 | 44.38 | 44.28 |
| 1. Moving or speaking too slowly or too fidgety | 30.10 | 13.66 | 28.58 | 30.35 | 29.99 | 30.74 |
| Anxiety Index | 59.20 | 24.15 | 55.61 | 59.24 | 60.55 | 59.61 |
| 1. Nervous when thinking about current circumstances | 66.54 | 23.06 | 64.42 | 61.77 | 74.50 | 66.13 |
| 1. Worried about one’s health | 59.98 | 22.26 | 59.17 | 60.99 | 58.40 | 60.68 |
| 1. Worried about family’s health | 61.07 | 23.13 | 57.67 | 60.56 | 62.55 | 62.15 |
| 1. Stressed about leaving the house | 77.59 | 26.21 | 71.86 | 75.44 | 81.72 | 79.63 |
| N | 48,434 | | 6,417 | 18,208 | 13,175 | 10,634 |
| **Sample after CEM** |  |  |  |  |  |  |
| Depression Index | 41.42 | 14.65 | 39.50 | 41.36 | 41.97 | 41.99 |
| 1. Little interest or pleasure in doing things | 43.72 | 21.92 | 43.42 | 44.88 | 42.48 | 43.48 |
| 1. Feeling down | 42.56 | 20.72 | 41.08 | 41.59 | 43.92 | 43.42 |
| 1. Trouble falling asleep or sleeping too much | 45.70 | 23.44 | 42.45 | 45.62 | 47.33 | 45.79 |
| 1. Feeling tired or having little energy | 48.60 | 22.11 | 48.41 | 49.05 | 48.82 | 47.68 |
| 1. Poor appetite or overeating | 41.32 | 22.37 | 39.10 | 41.28 | 41.44 | 42.56 |
| 1. Feeling bad about oneself | 36.33 | 19.68 | 34.37 | 35.57 | 37.21 | 37.74 |
| 1. Trouble concentrating on things | 42.96 | 22.72 | 38.59 | 42.46 | 44.58 | 44.48 |
| 1. Moving or speaking too slowly or too fidgety | 30.19 | 13.68 | 28.66 | 30.44 | 30.08 | 30.83 |
| Anxiety Index | 59.55 | 24.21 | 55.92 | 59.59 | 60.92 | 59.97 |
| 1. Nervous when thinking about current circumstances | 66.98 | 23.11 | 64.83 | 62.15 | 75.06 | 66.57 |
| 1. Worried about one’s health | 60.34 | 22.31 | 59.52 | 61.36 | 58.74 | 61.05 |
| 1. Worried about family’s health | 61.44 | 23.18 | 58.00 | 60.93 | 62.94 | 62.54 |
| 1. Stressed about leaving the house | 78.19 | 26.28 | 72.38 | 76.01 | 82.39 | 80.26 |
| N | 44,840 | | 5,874 | 17,172 | 12,060 | 9,734 |

Source: Own work using data from [https://COVID19-survey.org/results.html](https://covid19-survey.org/results.html)

For the computation of the descriptive statistics, individual sample weights have been used to correct for differences in income, education, age and gender structure between the general population of the country and the corresponding sample.

**Table A4. Individual characteristics by region**

|  | Inicial sample | | | | | Sample after CEM | | | | |
| --- | --- | --- | --- | --- | --- | --- | --- | --- | --- | --- |
|  | All sample | Eastern Europe | Northern Europe | Southern Europe | Western Europe | All sample | Eastern Europe | Northern Europe | Southern Europe | Western Europe |
| Gender |  |  |  |  |  |  |  |  |  |  |
| Man | 44.76 | 35.29 | 43.17 | 47.77 | 47.34 | 44.96 | 35.41 | 43.36 | 48.00 | 47.56 |
| Women | 54.30 | 64.31 | 55.75 | 51.67 | 51.65 | 54.59 | 64.72 | 56.06 | 51.94 | 51.92 |
| Other | 0.94 | 0.41 | 1.07 | 0.56 | 1.01 | 0.94 | 0.41 | 1.07 | 0.56 | 1.01 |
| Age | 41.15 | 34.22 | 43.93 | 41.34 | 39.71 | 41.32 | 34.34 | 44.12 | 41.51 | 39.87 |
|  | (12.65) | (10.05) | (12.68) | (12.87) | (12.27) | (12.63) | (10.04) | (12.66) | (12.85) | (12.25) |
| Number of years of education | 15.77 | 16.14 | 16.45 | 17.80 | 14.53 | 15.79 | 16.17 | 16.48 | 17.83 | 14.55 |
|  | (5.08) | (3.39) | (4.39) | (4.31) | (5.81) | (5.08) | (3.39) | (4.39) | (4.31) | (5.81) |
| Marital status |  |  |  |  |  |  |  |  |  |  |
| Married | 63.28 | 56.79 | 67.14 | 57.11 | 62.27 | 63.68 | 57.11 | 67.59 | 57.44 | 62.66 |
| Single | 36.72 | 43.21 | 32.86 | 42.89 | 37.73 | 36.85 | 43.40 | 32.97 | 43.07 | 37.87 |
| Household size |  |  |  |  |  | 0.00 | 0.00 | 0.00 | 0.00 | 0.00 |
| Living alone | 19.65 | 16.17 | 18.36 | 15.33 | 22.65 | 19.69 | 16.20 | 18.39 | 15.35 | 22.70 |
| 2 people | 34.25 | 31.56 | 35.44 | 31.08 | 34.53 | 34.37 | 31.66 | 35.57 | 31.18 | 34.65 |
| 3 people | 19.37 | 26.64 | 17.84 | 23.29 | 18.59 | 19.41 | 26.71 | 17.87 | 23.34 | 18.62 |
| More than 3 people | 26.73 | 25.64 | 28.36 | 30.30 | 24.23 | 26.80 | 25.71 | 28.44 | 30.39 | 24.29 |
| Specific country income quartile |  |  |  |  |  |  |  |  |  |  |
| 1^st^ quartile (lowest) | 25.84 | 26.28 | 25.28 | 29.74 | 25.29 | 25.91 | 26.35 | 25.34 | 29.83 | 25.35 |
| 2^nd^ quartile | 25.58 | 25.96 | 25.35 | 21.05 | 26.90 | 25.65 | 26.03 | 25.41 | 21.09 | 26.97 |
| 3^rd^ quartile | 24.90 | 25.23 | 24.61 | 25.02 | 25.08 | 24.96 | 25.29 | 24.67 | 25.08 | 25.14 |
| 4^th^ quartile (highest) | 23.69 | 22.53 | 24.75 | 24.19 | 22.72 | 23.75 | 22.58 | 24.81 | 24.25 | 22.77 |
| Has comorbidities ^A^ | 12.31 | 14.76 | 9.55 | 11.49 | 14.75 | 12.33 | 14.78 | 9.56 | 11.50 | 14.77 |
| Number of comorbidities (conditioned on having at least one) | 2.17 | 2.12 | 2.16 | 2.18 | 2.19 | 2.17 | 2.12 | 2.16 | 2.18 | 2.19 |
|  | (0.48) | (0.40) | (0.45) | (0.50) | (0.49) | (0.48) | (0.40) | (0.45) | (0.50) | (0.49) |
| N | 48,434 | 3,697 | 19,259 | 4,997 | 20,072 | 44,840 | 3,356 | 18,043 | 4,600 | 18,841 |
| % |  | 7.63 | 39.76 | 10.32 | 41.44 |  | 7.48 | 40.24 | 10.26 | 42.02 |

Source: Own work using data from [https://COVID19-survey.org/results.html](https://covid19-survey.org/results.html)

Standard errors between parenthesis.

^A^ Cardiovascular diseases, diabetes, hepatitis B, chronic obstructive pulmonary disease, chronic kidney diseases, and cancer.

Eastern Europe: Bulgaria, Czech Republic, Hungary, Romania, Slovakia and Ukraine.

Northern Europe: Denmark, Finland, Ireland, Norway, Sweden and United Kingdom.

Southern Europe: Greece, Italy, Portugal and Spain.

Western Europe: Austria, Belgium, France, Germany, Netherlands and Switzerland.

Income refers to monthly household income before taxes. Income quartiles have been computed for each country after adjusting by the square root of household size. Individual sample weights have been used to correct for differences in income, education, age and gender structure between the general population of the country and the corresponding sample.

**Table A5. Descriptive statistics by country**

|  | N | | | COVID cases per 1.000.000 inhab | | | Anxiety index | PHQ-8 Depression index_ | Stringency index |
| --- | --- | --- | --- | --- | --- | --- | --- | --- | --- |
|  | Initial sample | Sample after CEM | % | Confirmed | Recovered | Deceased |  |  |  |
| Austria | 1,074 | 980 | 91,25 | 486.22 | 9.76 | 2.97 | 60.06 | 41.22 | 84.79 |
|  |  |  |  | *220.05* | *30.91* | *3.28* | *22.76* | *13.83* | *0.00* |
| Belgium | 569 | 511 | 89,81 | 374.15 | 36.33 | 11.75 | 59.44 | 41.71 | 83.60 |
|  |  |  |  | *228.95* | *39.94* | *15.81* | *24.48* | *14.61* | *0.00* |
| Bulgaria | 329 | 295 | 89,67 | 33.38 | 0.96 | 0.71 | 60.18 | 45.03 | 75.02 |
|  |  |  |  | *10.55* | *0.75* | *0.29* | *26.09* | *16.43* | *0.11* |
| Czech Rep. | 267 | 247 | 92,51 | 135.53 | 1.02 | 0.51 | 56.41 | 41.85 | 79.27 |
|  |  |  |  | *62.69* | *1.29* | *0.95* | *25.19* | *14.53* | *1.43* |
| Denmark | 506 | 468 | 92,49 | 271.82 | 1.81 | 3.50 | 56.96 | 39.79 | 84.12 |
|  |  |  |  | *46.06* | *9.38* | *2.52* | *25.69* | *13.67* | *0.00* |
| Finland | 635 | 575 | 90,55 | 125.05 | 1.99 | 0.51 | 55.17 | 37.93 | 74.38 |
|  |  |  |  | *30.82* | *0.00* | *0.41* | *24.30* | *13.28* | *0.19* |
| France | 2,721 | 2,425 | 89,12 | 326.88 | 44.97 | 16.85 | 60.69 | 42.06 | 89.41 |
|  |  |  |  | *153.11* | *35.30* | *14.62* | *24.33* | *14.71* | *0.00* |
| Germany | 10,097 | 9,677 | 95,84 | 564.57 | 86.11 | 4.92 | 59.33 | 41.93 | 70.64 |
|  |  |  |  | *286.54* | *98.57* | *4.86* | *23.49* | *13.97* | *3.51* |
| Greece | 328 | 280 | 85,37 | 70.36 | 2.51 | 2.01 | 60.18 | 43.98 | 84.28 |
|  |  |  |  | *19.82* | *1.11* | *0.92* | *24.01* | *15.15* | *5.59* |
| Hungary | 239 | 224 | 93,72 | 15.90 | 1.64 | 0.77 | 62.22 | 44.54 | 74.42 |
|  |  |  |  | *9.86* | *0.87* | *0.41* | *24.59* | *14.06* | *1.59* |
| Ireland | 711 | 660 | 92,83 | 237.07 | 1.26 | 2.25 | 61.13 | 41.50 | 60.48 |
|  |  |  |  | *135.89* | *0.34* | *3.94* | *21.83* | *14.99* | *8.56* |
| Italy | 1,849 | 1,721 | 93,08 | 1,034.82 | 123.70 | 99.24 | 61.18 | 44.68 | 93.25 |
|  |  |  |  | *241.86* | *45.44* | *34.19* | *23.28* | *14.63* | *0.00* |
| Netherlands | 1,423 | 1,345 | 94,52 | 304.73 | 1.26 | 16.25 | 55.78 | 39.43 | 75.25 |
|  |  |  |  | *153.75* | *3.80* | *16.20* | *23.71* | *13.68* | *1.63* |
| Norway | 302 | 289 | 95,70 | 504.58 | 0.73 | 2.31 | 59.42 | 38.78 | 76.51 |
|  |  |  |  | *140.86* | *0.97* | *1.90* | *22.91* | *12.54* | *1.63* |
| Portugal | 550 | 511 | 92,91 | 217.23 | 1.24 | 3.02 | 65.92 | 43.18 | 62.57 |
|  |  |  |  | *142.58* | *1.34* | *3.57* | *22.98* | *16.96* | *0.00* |
| Romania | 801 | 747 | 93,26 | 28.41 | 3.53 | 0.38 | 64.16 | 40.90 | 80.22 |
|  |  |  |  | *16.21* | *1.42* | *0.64* | *23.33* | *15.01* | *2.17* |
| Slovakia | 609 | 529 | 86,86 | 35.25 | 1.32 | 0.20 | 58.63 | 41.12 | 82.14 |
|  |  |  |  | *3.19* | *0.40* | *0.05* | *24.39* | *13.79* | *0.00* |
| Spain | 2,270 | 2,088 | 91,98 | 817.85 | 88.53 | 56.81 | 62.87 | 40.73 | 78.07 |
|  |  |  |  | *342.84* | *83.92* | *33.95* | *26.01* | *14.65* | *2.20* |
| Sweden | 5,853 | 5,632 | 96,22 | 223.62 | 1.77 | 4.17 | 53.52 | 38.87 | 32.64 |
|  |  |  |  | *65.92* | *1.14* | *4.14* | *26.24* | *13.73* | *1.22* |
| Switzerland | 4,188 | 3,903 | 93,19 | 926.21 | 21.94 | 12.78 | 60.53 | 40.57 | 79.49 |
|  |  |  |  | *186.21* | *48.47* | *5.94* | *23.13* | *13.78* | *0.00* |
| Ukraine | 1,452 | 1,314 | 90,50 | 2.36 | 0.06 | 0.10 | 57.07 | 42.62 | 92.06 |
|  |  |  |  | *3.56* | *0.08* | *0.10* | *25.05* | *15.43* | *0.00* |
| United Kingdom | 11,252 | 10,419 | 92,60 | 102.31 | 1.12 | 5.37 | 61.78 | 42.16 | 47.32 |
|  |  |  |  | *61.84* | *0.34* | *5.47* | *23.30* | *15.73* | *18.23* |
| Eastern Europe | 3,697 | 3,356 | 90,78 | 26.67 | 1.27 | 0.31 | 59.43 | 42.29 | 84.28 |
|  |  |  |  | *38.41* | *1.55* | *0.48* | *24.80* | *15.07* | *6.72* |
| Northern Europe | 19,259 | 18,043 | 93,69 | 155.66 | 1.36 | 4.63 | 58.86 | 40.88 | 45.66 |
|  |  |  |  | *101.18* | *1.70* | *4.98* | *24.56* | *15.03* | *18.43* |
| Southern Europe | 4,997 | 4,600 | 92,06 | 782.96 | 86.29 | 62.99 | 62.40 | 42.67 | 82.39 |
|  |  |  |  | *412.16* | *75.76* | *45.84* | *24.61* | *15.05* | *9.99* |
| Western Europe | 20,072 | 18,841 | 93,87 | 579.79 | 55.63 | 9.07 | 59.55 | 41.45 | 76.48 |
|  |  |  |  | *313.56* | *81.83* | *10.00* | *23.57* | *14.04* | *7.30* |
| Total | 48,434 | 44,840 | 92,58 | 386.00 | 32.64 | 12.13 | 59.55 | 41.42 | 65.52 |
|  |  |  |  | *354.86* | *65.99* | *23.94* | *24.21* | *14.65* | *20.92* |

Source: Own work using data from: (i) [https://COVID19-survey.org/results.html](https://covid19-survey.org/results.html)for Depression Index (PHQ-8) and Anxiety Index; (ii) Coronavirus Pandemic Data Explorer <https://ourworldindata.org/coronavirus-data-explorer> for confirmed cases, recovered cases and deceased per 1,000,000 inhabitants (iii) [https://www.bsg.ox.ac.uk/research/research-projects/oxford-COVID-19-government-response-tracker](https://www.bsg.ox.ac.uk/research/research-projects/oxford-covid-19-government-response-tracker) for COVID-19 Government Response Stringency Index (Stringency Index).

Eastern Europe: Bulgaria, Czech Republic, Hungary, Romania, Slovakia and Ukraine. Northern Europe: Denmark, Finland, Ireland, Norway, Sweden and United Kingdom. Southern Europe: Greece, Italy, Portugal and Spain. Western Europe: Austria, Belgium, France, Germany, Netherlands and Switzerland.

Standard deviation in italics. Individual sample weights have been used to correct for differences in income, education, age and gender structure between the general population of the country and the corresponding sample.

**Table A6. Descriptive statistics by country. Continuation**

|  | Items for Depression Index (PHQ-8) | | | | | | | | Items for Anxiety Index | | | |
| --- | --- | --- | --- | --- | --- | --- | --- | --- | --- | --- | --- | --- |
|  | Little interest in things | Feeling down | Sleeping problems | Feeling tired | Appetite problems | Feeling bad about oneself | Trouble concentr. | Speaking problems | Nervous and restless | Worried about one’s health | Worried about family’s health | Stressed leaving house |
| Austria | 44.63 | 41.77 | 44.64 | 47.76 | 40.95 | 35.15 | 43.67 | 30.82 | 59.62 | 61,43 | 58,59 | 71,94 |
|  | *20.86* | *19.94* | *21.53* | *20.14* | *22.01* | *18.47* | *21.81* | *14.17* | *23.07* | *20,73* | *23,09* | *22,09* |
| Belgium | 43.04 | 41.87 | 45.24 | 49.94 | 41.65 | 36.51 | 44.62 | 30.49 | 64.31 | 61,93 | 59,54 | 77,67 |
|  | *21.40* | *20.06* | *23.76* | *22.86* | *22.40* | *19.13* | *22.47* | *14.42* | *22.24* | *21,88* | *22,94* | *22,17* |
| Bulgaria | 48.92 | 50.02 | 47.80 | 52.79 | 43.96 | 38.60 | 46.11 | 31.63 | 72.18 | 59,50 | 62,33 | 85,90 |
|  | *25.31* | *24.87* | *25.02* | *24.12* | *24.27* | *22.64* | *23.93* | *16.34* | *24.15* | *24,94* | *25,75* | *22,44* |
| Czech Rep. | 46.14 | 42.88 | 43.92 | 48.74 | 42.88 | 37.87 | 42.97 | 29.01 | 64.23 | 61,12 | 59,68 | 73,41 |
|  | *23.26* | *20.90* | *24.31* | *22.74* | *22.75* | *20.53* | *21.88* | *11.42* | *22.03* | *20,47* | *22,88* | *22,97* |
| Denmark | 43.44 | 39.08 | 42.72 | 48.50 | 39.08 | 35.40 | 40.08 | 29.64 | 66.88 | 65,56 | 59,92 | 75,11 |
|  | *22.85* | *18.95* | *22.30* | *23.13* | *21.53* | *19.32* | *22.13* | *12.16* | *20.38* | *21,38* | *24,38* | *22,22* |
| Finland | 39.64 | 38.77 | 39.44 | 44.89 | 37.58 | 33.69 | 40.88 | 28.27 | 60.91 | 64,19 | 61,25 | 73,16 |
|  | *20.52* | *18.97* | *20.08* | *20.83* | *19.87* | *17.43* | *21.99* | *10.92* | *21.81* | *21,91* | *21,64* | *22,26* |
| France | 42.69 | 41.75 | 46.78 | 48.29 | 43.56 | 37.98 | 44.41 | 30.66 | 64.65 | 61,05 | 59,56 | 77,68 |
|  | *21.48* | *20.20* | *23.92* | *21.94* | *23.56* | *20.86* | *23.35* | *14.76* | *22.98* | *21,99* | *23,61* | *21,93* |
| Germany | 45.45 | 42.16 | 46.48 | 49.90 | 41.07 | 35.61 | 43.49 | 30.96 | 60.00 | 61,67 | 60,35 | 73,85 |
|  | *20.90* | *19.66* | *22.71* | *21.01* | *21.47* | *18.92* | *21.87* | *14.07* | *22.64* | *20,94* | *22,58* | *22,48* |
| Greece | 51.11 | 49.71 | 44.02 | 47.55 | 45.64 | 37.94 | 42.94 | 32.42 | 65.48 | 58,94 | 60,54 | 82,94 |
|  | *24.52* | *23.46* | *23.81* | *21.52* | *24.28* | *21.73* | *23.16* | *15.66* | *23.78* | *24,78* | *23,70* | *21,86* |
| Hungary | 48.20 | 51.41 | 45.28 | 52.57 | 42.44 | 40.96 | 45.82 | 29.16 | 68.49 | 55,96 | 59,00 | 87,79 |
|  | *22.62* | *21.33* | *23.53* | *22.39* | *21.61* | *22.36* | *24.24* | *11.31* | *23.07* | *25,29* | *24,48* | *19,38* |
| Ireland | 41.73 | 42.16 | 45.81 | 47.23 | 43.23 | 37.34 | 44.32 | 29.87 | 74.47 | 60,99 | 64,29 | 85,21 |
|  | *21.84* | *21.00* | *23.47* | *22.19* | *24.14* | *20.16* | *23.18* | *13.62* | *22.32* | *21,40* | *21,43* | *20,73* |
| Italy | 47.23 | 47.81 | 49.01 | 49.56 | 45.79 | 38.51 | 47.19 | 31.91 | 66.24 | 56,38 | 60,06 | 76,48 |
|  | *21.42* | *20.48* | *24.39* | *21.09* | *24.07* | *20.02* | *23.27* | *15.44* | *21.89* | *23,32* | *21,11* | *21,97* |
| Netherlands | 40.78 | 39.31 | 42.98 | 46.54 | 38.18 | 33.95 | 44.37 | 29.03 | 65.27 | 62,55 | 58,11 | 75,74 |
|  | *21.09* | *19.16* | *22.18* | *21.80* | *20.53* | *17.13* | *23.93* | *12.04* | *21.29* | *21,90* | *23,60* | *22,23* |
| Norway | 41.30 | 40.14 | 42.98 | 47.75 | 39.14 | 34.23 | 36.98 | 27.39 | 66.06 | 61,90 | 59,96 | 68,95 |
|  | *20.31* | *20.03* | *22.09* | *21.17* | *19.40* | *17.44* | *19.01* | *8.58* | *17.55* | *19,03* | *24,57* | *18,90* |
| Portugal | 44.62 | 44.65 | 46.72 | 46.72 | 47.13 | 38.09 | 44.05 | 33.11 | 72.77 | 58,49 | 65,92 | 89,00 |
|  | *22.44* | *21.99* | *23.92* | *23.09* | *25.07* | *21.90* | *23.18* | *17.16* | *23.06* | *23,37* | *21,64* | *19,03* |
| Romania | 46.41 | 43.35 | 44.26 | 44.52 | 43.32 | 34.83 | 38.91 | 31.12 | 69.33 | 58,32 | 62,71 | 81,27 |
|  | *23.70* | *21.54* | *23.01* | *21.15* | *23.19* | *19.45* | *20.58* | *14.56* | *21.47* | *23,32* | *22,44* | *22,48* |
| Slovakia | 46.74 | 38.11 | 41.75 | 48.67 | 39.94 | 42.87 | 42.87 | 27.58 | 65.15 | 59,20 | 59,63 | 83,61 |
|  | *23.41* | *19.12* | *21.94* | *22.21* | *21.87* | *21.79* | *22.68* | *10.59* | *21.54* | *23,26* | *22,95* | *20,97* |
| Spain | 43.04 | 40.59 | 44.09 | 43.67 | 42.60 | 36.28 | 44.06 | 31.16 | 70.51 | 63,60 | 62,95 | 83,47 |
|  | *21.91* | *19.30* | *22.47* | *20.24* | *22.21* | *19.01* | *23.20* | *14.63* | *22.34* | *23,17* | *23,06* | *21,06* |
| Sweden | 42.51 | 40.50 | 41.44 | 49.50 | 37.39 | 33.81 | 37.40 | 28.04 | 62.54 | 59,73 | 56,49 | 68,65 |
|  | *22.79* | *20.62* | *21.92* | *22.65* | *20.35* | *18.08* | *20.00* | *10.57* | *21.53* | *20,73* | *24,59* | *22,22* |
| Switzerland | 42.60 | 40.80 | 45.62 | 48.40 | 42.18 | 34.41 | 40.26 | 29.96 | 65.07 | 60,37 | 62,52 | 80,25 |
|  | *21.52* | *19.78* | *22.83* | *21.55* | *22.30* | *17.63* | *21.17* | *12.93* | *21.83* | *21,87* | *22,11* | *21,51* |
| Ukraine | 40.05 | 49.26 | 45.42 | 49.71 | 41.09 | 42.66 | 41.60 | 30.91 | 66.29 | 61,39 | 76,67 | 90,46 |
|  | *22.30* | *23.79* | *24.86* | *24.42* | *23.41* | *24.34* | *23.50* | *14.71* | *23.21* | *24,15* | *23,86* | *18,87* |
| United Kingdom | 41.63 | 43.86 | 48.13 | 48.59 | 41.70 | 37.40 | 45.40 | 30.22 | 77.00 | 58,54 | 63,85 | 83,85 |
|  | *21.98* | *21.58* | *24.82* | *23.12* | *23.14* | *20.64* | *24.24* | *14.15* | *22.25* | *23,61* | *22,28* | *21,33* |
| Eastern Europe | 44.28 | 45.89 | 44.66 | 48.81 | 41.85 | 40.18 | 42.00 | 30.22 | 67.29 | 59,82 | 67,19 | 85,53 |
|  | *23.41* | *22.76* | *23.93* | *23.23* | *23.06* | *22.60* | *22.82* | *13.87* | *22.66* | *23,77* | *24,75* | *21,28* |
| Northern Europe | 41.87 | 42.43 | 45.50 | 48.67 | 40.21 | 36.08 | 42.51 | 29.43 | 71.54 | 59,42 | 61,38 | 78,46 |
|  | *22.18* | *21.17* | *23.89* | *22.86* | *22.26* | *19.77* | *23.07* | *12.96* | *22.94* | *22,56* | *23,28* | *22,71* |
| Southern Europe | 45.29 | 44.30 | 46.19 | 46.44 | 44.48 | 37.41 | 45.14 | 31.73 | 68.85 | 60,06 | 62,05 | 81,45 |
|  | *22.10* | *20.64* | *23.55* | *21.13* | *23.43* | *19.93* | *23.27* | *15.30* | *22.48* | *23,59* | *22,32* | *21,64* |
| Western Europe | 44.04 | 41.59 | 45.96 | 49.02 | 41.44 | 35.57 | 43.04 | 30.55 | 62.16 | 61,37 | 60,42 | 75,85 |
|  | *21.19* | *19.76* | *22.85* | *21.34* | *21.96* | *18.83* | *22.14* | *13.83* | *22.57* | *21,37* | *22,77* | *22,32* |
| Total | 43.34 | 42.56 | 45.70 | 48.60 | 41.32 | 36.33 | 42.96 | 30.19 | 66.98 | 60,34 | 61,44 | 78,19 |
|  | *21.92* | *20.72* | *23.44* | *22.11* | *22.37* | *19.68* | *22.72* | *13.68* | *23.11* | *22,31* | *23,18* | *22,50* |

Source: Own work using data from[https://COVID19-survey.org/results.html](https://covid19-survey.org/results.html) . Standard deviation in italics.

Items for PHQ-8 Depression Index and for Anxiety Index take values between 0 and 100.

Eastern Europe: Bulgaria, Czech Republic, Hungary, Romania, Slovakia and Ukraine. Northern Europe: Denmark, Finland, Ireland, Norway, Sweden and United Kingdom. Southern Europe: Greece, Italy, Portugal and Spain. Western Europe: Austria, Belgium, France, Germany, Netherlands and Switzerland.

Individual sample weights have been used to correct for differences in income, education, age and gender structure between the general population of the country and the corresponding sample.

**Appendix B**

**Figure B1. Event study results. Effect of the days before/after pandemic reached category 5 and interaction between days before/after pandemic of category 5 and lockdown over Depression Index and Anxiety Index.**

|  |  |
| --- | --- |
|  |  |

Upper graphs show the estimated coefficients for $\sum_{j=-7}^{j=7} \delta_{0k}D_{kc}P$ of equation 2 for Depression Index (left) and Anxiety Index (right). Lower graphs show the estimated coefficients for $\sum_{j=-7}^{j=7} \delta_{2k}D_{kc}L_{ct}P_{ct}$ of equation 2 for Depression Index (left) and Anxiety Index (right). See Table 5 for the detail of coefficients and standard deviations.

Red dashed line used to signal the day when pandemic reached category 5 according to the Pandemic Severity Index.

**Figure B2. Relationship between R-squared and model specification**

|  |  |
| --- | --- |
|  |  |

This ﬁgure shows 4 different scatterplots. In the y-axis, we represent the absolute value of the lockdown coeﬃcient (upper) and interaction between lockdown and pandemic of category 5 (lower) (y-axis). In the x-axis we represent the model R-squared (x-axis). Dependent variables is Depression Index (left figures) and Anxiety Index (right figures). The absolute value of the lockdown coeﬃcient (or the interaction between lockdown and pandemic of category 5) and the R-squared result of the respective outcome on different combinations among the explanatory variables (sex, age, years of education, marital status, income, number of household members, comorbidities, country fixed effects and day fixed effects). The maroon line represents a locally weighted regression Robust standard errors are obtained at the day level. The specification reported in the paper (M5) is coloured in red.

**Table B1. Event study results. Estimated coefficients for days before/after pandemic reached category 5 and interaction between day before/after pandemic reached category 5 and lockdown.**

|  | Depression Index | | Anxiety Index | |
| --- | --- | --- | --- | --- |
|  | Effect of days before/after pandemic reached category 5 | Effect of days before/after pandemic reached category 5 and lockdown | Effect of days before/after pandemic reached category 5 | Effect of days before/after pandemic reached category 5 and lockdown |
| Day -7 | 0.153 | -2.575 | -3.614 | -2.459 |
|  | (5.926) | (3.572) | (5.715) | (3.603) |
| Day -6 | 11.021 | -9.025** | 11.343** | -9.104** |
|  | (6.032) | (3.644) | (5.820) | (3.675) |
| Day -5 | -4.472* | 2.523 | 3.991 | 2.626** |
|  | (2.112) | (1.278) | (2.030) | (1.298) |
| Day -4 | 0.229 | -0.256 | 0.911 | -0.040 |
|  | (1.623) | (0.985) | (1.562) | (1.167) |
| Day -3 | -2.217 | 1.227 | -4.231 | 1.075 |
|  | (2.337) | (1.420) | (2.255) | (1.704) |
| Day -2 | -7.766 | 0.738 | -3.263 | 2.523 |
|  | (3.489) | (2.112) | (3.365) | (2.685) |
| Day -1 | -8.510** | 0.981 | -2.206 | 0.781 |
|  | (3.520) | (2.132) | (3.396) | (2.952) |
| Day pandemic reached Cat. 5 | 0.893*** | -1.622*** | 8.220*** | -1.599*** |
|  | (0.331) | (0.331) | (0.592) | (0.351) |
| Day +1 | 3.766*** | -1.922*** | 7.299*** | -2.239*** |
|  | (0.723) | (0.441) | (0.692) | (0.451) |
| Day +2 | 3.326*** | -1.684*** | 10.769*** | -1.643*** |
|  | (0.481) | (0.290) | (0.461) | (0.300) |
| Day +3 | 2.735*** | -1.971*** | 9.929*** | -2.567*** |
|  | (0.763) | (0.471) | (0.743) | (0.481) |
| Day +4 | 1.535 | -0.484 | 3.791*** | -2.350 |
|  | (1.126) | (0.682) | (1.086) | (0.793) |
| Day +5 | 0.461 | -0.298 | 1.995 | -0.690 |
|  | (1.714) | (1.035) | (1.643) | (1.217) |
| Day +6 | -1.623 | 1.240 | 2.636 | 1.209 |
|  | (2.163) | (1.319) | (2.081) | (1.471) |
| Day +7 | -2.127 | 1.570 | 4.301 | 1.159 |
|  | (2.449) | (1.481) | (1.126) | (1.552) |

All models include the following explanatory variables: man, other gender (omitted: women), age and its square, number of years of education and its square, married (omitted: single), specific-country quartile income (omitted: lowest quartile), number of household members (omitted: living alone), number of comorbidities, country fixed effects, day fixed effects. Individual sample weights have been used to correct for differences in income, education, age and gender structure between the general population of the country and the corresponding sample. Robust standard errors. ***, ** and * denote statistical signiﬁcance at the 1%, 5% and 10% level.

**Appendix C**

**Table C1. Difference in difference model. Items of the PHQ-8 Depression Index**

|  | Item 1: Little interest or pleasure in doing things | | | | | Item 2: Feeling down | | | | |
| --- | --- | --- | --- | --- | --- | --- | --- | --- | --- | --- |
|  | M1 | M2 | M3 | M4 | M5 | M1 | M2 | M3 | M4 | M5 |
| Lockdown | 0.012 | -0.026 | -0.037 | -0.046 | -0.039 | -0.113 | -0.109 | -0.164 | -0.110 | -0.123 |
|  | (0.327) | (0.327) | (0.327) | (0.327) | (0.327) | (0.306) | (0.305) | (0.305) | (0.305) | (0.305) |
| Pandemic_cat5 | -1.118*** | -1.000*** | -1.016*** | -1.01*** | -1.019*** | 3.366 | 3.420 | 3.406 | 3.477 | 3.403 |
|  | (0.333) | (0.333) | (0.333) | (0.333) | (0.333) | (0.311) | (0.311) | (0.311) | (0.310) | (0.311 |
| Lockdown&Pand_cat5 | -0.553 | -0.711 | -0.651 | -0.623 | -0.594 | -2.012 | -2.082 | -2.032 | -2.000 | -1.970) |
|  | (0.433) | (0.433) | (0.433) | (0.433) | (0.433) | (0.405) | (0.404) | (0.405) | (0.404) | (0.404 |
| Constant | 67.921*** | 66.185*** | 66.511*** | 65.976*** | 65.669*** | 67.288*** | 65.101*** | 65.607*** | 64.970*** | 64.645*** |
|  | (1.257) | (1.287) | (1.327) | (1.325) | (1.329) | (1.172) | (1.201) | (1.238) | (1.235) | (1.239) |
| N | 44,840 | 44,840 | 44,840 | 44,840 | 44,840 | 44,840 | 44,840 | 44,840 | 44,840 | 44,840 |
| R^2^ | 0.232 | 0.238 | 0.238 | 0.242 | 0.240 | 0.257 | 0.262 | 0.262 | 0.267 | 0.265 |
| F | 55.525 | 63.573 | 57.991 | 60.004 | 56.474 | 108.769 | 111.664 | 101.471 | 104.872 | 97.745 |
| p | 0.000 | 0.000 | 0.000 | 0.000 | 0.000 | 0.000 | 0.000 | 0.000 | 0.000 | 0.000 |
|  | Item 3: Trouble falling asleep or sleeping too much | | | | | Item 4: Feeling tired or having little energy | | | | |
|  | M1 | M2 | M3 | M4 | M5 | M1 | M2 | M3 | M4 | M5 |
| Lockdown | 3.363*** | 3.326*** | 3.288*** | 2.946*** | 3.324*** | -0.272 | -0.334 | -0.338 | -0.666 | -0.307 |
|  | (0.350) | (0.350) | (0.350) | (0.349) | (0.350) | (0.328) | (0.328) | (0.328) | (0.327) | (0.328) |
| Pandemic_cat5 | 5.934*** | 6.056*** | 6.035*** | 6.132*** | 6.029*** | 0.704*** | 0.834*** | 0.848*** | 0.943*** | 0.848*** |
|  | (0.357) | (0.357) | (0.357) | (0.355) | (0.356) | (0.334) | (0.335) | (0.335) | (0.333) | (0.334) |
| Lockdown&Pand_cat5 | -5.472*** | -5.600*** | -5.547*** | -5.510*** | -5.510*** | -2.298*** | -2.451*** | -2.479*** | -2.434*** | -2.420*** |
|  | (0.463) | (0.463) | (0.464) | (0.462) | (0.464) | (0.434) | (0.435) | (0.435) | (0.433) | (0.435) |
| Constant | 59.475*** | 57.946*** | 57.901*** | 57.131*** | 57.272*** | 68.769*** | 68.672*** | 69.291*** | 68.354*** | 68.413*** |
|  | (1.347) | (1.380) | (1.423) | (1.418) | (1.425) | (1.261) | (1.293) | (1.333) | (1.327) | (1.335) |
| N | 44,840 | 44,840 | 44,840 | 44,840 | 44,840 | 44,840 | 44,840 | 44,840 | 44,840 | 44,840 |
| R^2^ | 0.232 | 0.236 | 0.236 | 0.243 | 0.238 | 0.244 | 0.246 | 0.246 | 0.255 | 0.248 |
| F | 55.526 | 60.112 | 54.807 | 62.408 | 52.143 | 80.363 | 79.072 | 72.027 | 83.085 | 68.911 |
| p | 0.000 | 0.000 | 0.000 | 0.000 | 0.000 | 0.000 | 0.000 | 0.000 | 0.000 | 0.000 |
|  | Item 5: Poor appetite or overeating | | | | | Item 6: Feeling bad about oneself | | | | |
|  | M1 | M2 | M3 | M4 | M5 | M1 | M2 | M3 | M4 | M5 |
| Lockdown | 1.919*** | 1.825*** | 1.819*** | 1.526*** | 1.847*** | 0.437 | 0.440 | 0.469 | 0.477 | 0.519 |
|  | (0.331) | (0.330) | (0.331) | (0.330) | (0.331) | (0.292) | (0.292) | (0.292) | (0.292) | (0.291) |
| Pandemic_cat5 | 3.119*** | 3.332*** | 3.344*** | 3.427*** | 3.342*** | 3.038*** | 3.094*** | 3.098*** | 3.161*** | 3.093*** |
|  | (0.337) | (0.337) | (0.337) | (0.336) | (0.337) | (0.298) | (0.297) | (0.298) | (0.297) | (0.297) |
| Lockdown&Pand_cat5 | -2.341*** | -2.584*** | -2.598*** | -2.562*** | -2.559*** | -1.510*** | -1.584*** | -1.622*** | -1.594*** | -1.555*** |
|  | (0.438) | (0.438) | (0.438) | (0.437) | (0.438) | (0.386) | (0.386) | (0.386) | (0.385) | (0.385) |
| Constant | 59.374*** | 58.678*** | 59.247*** | 58.530*** | 58.641*** | 59.184*** | 57.064*** | 56.545*** | 55.993*** | 55.520*** |
|  | (1.273) | (1.302) | (1.343) | (1.338) | (1.345) | (1.121) | (1.148) | (1.184) | (1.181) | (1.184) |
| N | 44,840 | 44,840 | 44,840 | 44,840 | 44,840 | 44,840 | 44,840 | 44,840 | 44,840 | 44,840 |
| R^2^ | 0.249 | 0.256 | 0.256 | 0.262 | 0.257 | 0.243 | 0.249 | 0.249 | 0.254 | 0.253 |
| F | 91.047 | 98.815 | 89.581 | 94.696 | 83.574 | 79.344 | 84.700 | 77.026 | 80.161 | 77.325 |
| p | 0.000 | 0.000 | 0.000 | 0.000 | 0.000 | 0.000 | 0.000 | 0.000 | 0.000 | 0.000 |
|  | Item 7: Trouble concentrating on things | | | | | Item 8: Moving or speaking too slowly or too fidgety | | | | |
|  | M1 | M2 | M3 | M4 | M5 | M1 | M2 | M3 | M4 | M5 |
| Lockdown | 3.503*** | 3.671*** | 3.566*** | 3.356*** | 3.606*** | 1.264*** | 1.247*** | 1.248*** | 1.073*** | 1.268*** |
|  | (0.335) | (0.334) | (0.335) | (0.335) | (0.334) | (0.205) | (0.205) | (0.205) | (0.205) | (0.205) |
| Pandemic_cat5 | 6.762*** | 6.578*** | 6.532*** | 6.591*** | 6.523*** | 1.429 | 1.468*** | 1.450*** | 1.500*** | 1.448*** |
|  | (0.342) | (0.341) | (0.341) | (0.340) | (0.341) | (0.209) | (0.209) | (0.209) | (0.209) | (0.209) |
| Lockdown&Pand_cat5 | -4.974*** | -4.817*** | -4.682*** | -4.659*** | -4.656*** | -1.248 | -1.296*** | -1.277*** | -1.253*** | -1.255*** |
|  | (0.444) | (0.443) | (0.443) | (0.443) | (0.443) | (0.272) | (0.272) | (0.273) | (0.272) | (0.273) |
| Constant | 64.265*** | 58.651*** | 59.033*** | 58.552*** | 58.531*** | 42.174*** | 41.904*** | 41.100*** | 40.633*** | 40.769*** |
|  | (1.289) | (1.318) | (1.358) | (1.357) | (1.361) | (0.786) | (0.807) | (0.832) | (0.830) | (0.834) |
| N | 44,840 | 44,840 | 44,840 | 44,840 | 44,840 | 44,840 | 44,840 | 44,840 | 44,840 | 44,840 |
| R^2^ | 0.254 | 0.262 | 0.263) | 0.266 | 0.264 | 0.219 | 0.220 | 0.220 | 0.228 | 0.222 |
| F | 102.138 | 111.861 | 103.039 | 101.714 | 96.382 | 33.662 | 33.075 | 30.561 | 38.334 | 29.776 |
| p | 0.000 | 0.000 | 0.000 | 0.000 | 0.000 | 0.000 | 0.000 | 0.000 | 0.000 | 0.000 |

Lockdown is a binary variable that takes the value one from the day the lockdown becomes effective, and 0 before.

Pandemic category 5: is a binary variable if the case fatality rate is higher or equal than 2 per cent. The case fatality rate is the percentage of deceased with respect to confirmed cases. The category 5 corresponds to the highest level of the Pandemic Severity Index. <https://www.cdc.gov/media/pdf/mitigationslides.pdf>

M1 includes lockdown, pandemic of category 5, interaction between lockdown and pandemic of category 5, day fixed effects and country fixed effects. M2 includes the same explanatory variables than M1 and also male, other gender (omitted: women), age and its squared. M3 includes the same explanatory variables than M2 and also married (omitted: single), years of education and number of household members (omitted: living alone). M4 includes the same explanatory variables than M3 and also having any comorbidity and number of comorbidities. M5 includes the same explanatory variables than M4 and also household income quartile (omitted: lowest quartile). Individual sample weights have been used to correct for differences in income, education, age and gender structure between the general population of the country and the corresponding sample. Robust standard errors. ***, ** and * denote statistical signiﬁcance at the 1%, 5% and 10% level.

|  |  |  |  |  |  |  |  |  |  |
| --- | --- | --- | --- | --- | --- | --- | --- | --- | --- |
|  |  |  |  |  |  |  |  |  |  |

**Table C2. Difference in difference model. Items of the Anxiety Index**

|  | Item 1: Nervous when thinking about current circumstances | | | | | Item 2: Worried about one’s health | | | | | |  |
| --- | --- | --- | --- | --- | --- | --- | --- | --- | --- | --- | --- | --- |
|  | M1 | M2 | M3 | M4 | M5 | | M1 | M2 | M3 | M4 | M5 | |
| Lockdown | -0.588 | -0.499 | -0.428 | -0.536 | -0.417 | | 0.830*** | 0.864*** | 0.850*** | 0.852*** | 0.840*** | |
|  | (0.336) | (0.336) | (0.337) | (0.336) | (0.335) | | (0.335) | (0.335) | (0.336) | (0.335) | (0.335) | |
| Pandemic_cat5 | 12.928*** | 12.738*** | 12.772*** | 12.807*** | 12.770*** | | -1.432*** | -1.466*** | -1.474*** | -1.502*** | -1.474 | |
|  | (0.343) | (0.343) | (0.343) | (0.343) | (0.341) | | (0.340) | (0.342) | (0.342) | (0.342) | (0.341) | |
| Lockdown&Pand_cat5 | -7.157*** | -6.997*** | -7.083*** | -7.072*** | -7.072*** | | 1.133*** | 1.171*** | 1.202*** | 1.185*** | 1.186*** | |
|  | (0.444) | (0.444) | (0.444) | (0.444) | (0.442) | | (0.442) | (0.443) | (0.443) | (0.443) | (0.444) | |
| Constant | 44.211*** | 47.432*** | 46.707*** | 46.227*** | 46.241*** | | 44.430*** | 46.446*** | 46.477*** | 47.602*** | 47.476*** | |
|  | (1.298) | (1.337) | (1.337) | (1.340) | (1.261) | | (1.294) | (1.333) | (1.333) | (1.336) | (1.354) | |
| N | 44,840 | 44,840 | 44,840 | 44,840 | 44,840 | | 44,840 | 44,840 | 44,840 | 44,840 | 44,840 | |
| R^2^ | 0.273 | 0.274 | 0.274 | 0.275 | 0.275 | | 0.210 | 0.211 | 0.211 | 0.212 | 0.211 | |
| F | 94.666 | 78.134 | 80.244 | 83.917 | 81.981 | | 80.170 | 89.538 | 87.717 | 88.285 | 86.417 | |
| p | 0.000 | 0.000 | 0.000 | 0.000 | 0.000 | | 0.000 | 0.000 | 0.000 | 0.000 | 0.000 | |
|  | Item 3: Worried about family’s health | | | | | Item 4: Stressed about leaving the house | | | | | |  |
|  | M1 | M2 | M3 | M4 | M5 | | M1 | M2 | M3 | M4 | M5 | |
| Lockdown | 4.298*** | 4.278*** | 4.243*** | 3.800*** | 4.257*** | | 5.400*** | 5.432*** | 5.422*** | 5.194*** | 5.441*** | |
|  | (0.348) | (0.348) | (0.347) | (0.348) | (0.331) | | (0.332) | (0.332) | (0.332) | (0.332) | (0.332) | |
| Pandemic_cat5 | 6.006*** | 6.022*** | 6.009*** | 6.130*** | 6.007*** | | 12.360*** | 12.304*** | 12.234*** | 12.299*** | 12.234*** | |
|  | (0.355) | (0.355) | (0.353) | (0.355) | (0.338) | | (0.338) | (0.338) | (0.338) | (0.338) | (0.338) | |
| Lockdown&Pand_cat5 | -4.028*** | -4.040*** | -4.016*** | -3.973*** | -4.001*** | | -7.117*** | -7.069*** | -7.033*** | -7.014*** | -7.002*** | |
|  | (0.460) | (0.460) | (0.458) | (0.460) | (0.437) | | (0.438) | (0.438) | (0.438) | (0.438) | (0.439) | |
| Constant | 59.946*** | 51.618*** | 51.719*** | 60.036*** | 61.232*** | | 76.037*** | 75.214*** | 79.235*** | 78.346*** | 70.140*** | |
|  | (1.343) | (1.384) | (1.377) | (1.387) | (1.249) | | (1.282) | (1.320) | (1.319) | (1.323) | (1.325) | |
| N | 44,840 | 44,840 | 44,840 | 44,840 | 44,840 | | 44,840 | 44,840 | 44,840 | 44,840 | 44,840 | |
| R^2^ | 0.211 | 0.211 | 0.211 | 0.222 | 0.212 | | 0.247 | 0.247 | 0.248 | 0.251 | 0.249 | |
| F | 22.220 | 18.287 | 19.288 | 39.875 | 17.854 | | 43.390 | 30.745 | 40.357 | 50.835 | 41.237 | |
| p | 0.000 | 0.000 | 0.000 | 0.000 | 0.000 | | 0.000 | 0.000 | 0.000 | 0.000 | 0.000 | |

Lockdown is a binary variable that takes the value one from the day the lockdown becomes effective, and 0 before.

Pandemic category 5: is a binary variable if the case fatality rate is higher or equal than 2 per cent. The case fatality rate is the percentage of deceased with respect to confirmed cases. The category 5 corresponds to the highest level of the Pandemic Severity Index. <https://www.cdc.gov/media/pdf/mitigationslides.pdf>

M1 includes lockdown, pandemic of category 5, interaction between lockdown and pandemic of category 5, day fixed effects and country fixed effects. M2 includes the same explanatory variables than M1 and also male, other gender (omitted: women), age and its squared. M3 includes the same explanatory variables than M2 and also married (omitted: single), years of education and number of household members (omitted: living alone). M4 includes the same explanatory variables than M3 and also having any comorbidity and number of comorbidities. M5 includes the same explanatory variables than M4 and also household income quartile (omitted: lowest quartile). Individual sample weights have been used to correct for differences in income, education, age and gender structure between the general population of the country and the corresponding sample. Robust standard errors. ***, ** and * denote statistical signiﬁcance at the 1%, 5% and 10% level.

**Table C3. Heterogeneity in the difference in difference model for Depression Index and Anxiety Index**

|  | PHQ-8 Depression Index | | | | | Anxiety Index | | | | | |
| --- | --- | --- | --- | --- | --- | --- | --- | --- | --- | --- | --- |
| **Age** | <=30 years | 31-40 years | 41-50 years | 51-60 years | >60 years | <=30 years | 31-40 years | 41-50 years | 51-60 years | >60 years |  |
| Lockdown | 0.304 | 0.322 | 1.895*** | 2.794*** | 1.884*** | 1.7 | 1.726*** | 6.740*** | 6.617*** | 6.837*** |  |
|  | (0.607) | (0.388) | (0.391) | (0.487) | (0.626) | (0.950) | (0.648) | (0.686) | (0.898) | (1.283) |  |
| Pandemic_cat5 | 2.014*** | 2.408*** | 3.317*** | 3.639*** | 2.350 | 4.473*** | 2.913*** | 7.839*** | 7.318*** | 7.578*** |  |
|  | (0.647) | (0.412) | (0.393) | (0.471) | (0.570) | (1.011) | (0.686) | (0.690) | (0.868) | (1.170) |  |
| Lockdown&Pand_cat5 | -1.444*** | -1.645*** | -3.552*** | -3.818*** | -2.995*** | -3.992*** | -2.357*** | -6.033*** | -5.607*** | -7.993*** |  |
|  | (0.744) | (0.527) | (0.543) | (0.654) | (0.827) | (1.163) | (0.877) | (0.952) | (1.204) | (1.691) |  |
| Constant | 60.277*** | 62.399*** | 64.200*** | 67.047*** | 64.387*** | 60.744*** | 61.327*** | 61.245*** | 61.224*** | 62.727*** |  |
|  | (7.737) | (16.253) | (22.955) | (12.030) | (10.675) | (11.506) | (22.474) | (23.357) | (21.962) | (18.789) |  |
| N | 9,932 | 12,976 | 10,360 | 6,812 | 3,511 | 9,932 | 12,976 | 10,360 | 6,812 | 3,511 |  |
| R^2^ | 0.057 | 0.046 | 0.045 | 0.050 | 0.045 | 0.017 | 0.012 | 0.019 | 0.025 | 0.023 |  |
| F | 21.853 | 25.586 | 19.155 | 13.139 | 5.981 | 5.434 | 5.476 | 7.092 | 5.912 | 2.894 |  |
| p | 0.000 | 0.000 | 0.000 | 0.000 | 0.000 | 0.000 | 0.000 | 0.000 | 0.000 | 0.000 |  |
|  | PHQ-8 Depression Index | | | | | Anxiety Index | | | | | |
| **Years of education** | <=5 y  ears | 6-10  years | 11-15 years | 16-20 years | >20  years | <=5 y  ears | 6-10  years | 11-15 years | 16-20 years | >20  years |  |
| Lockdown | 1.181 | 1.424*** | 2.846*** | 1.558*** | 2.885*** | 6.364*** | 3.821*** | 3.918*** | 5.226*** | 2.790*** |  |
|  | (0.846) | (0.290) | (0.913) | (0.437) | (0.708) | (1.484) | (0.506) | (1.607) | (0.724) | (1.206) |  |
| Pandemic_cat5 | 1.039 | 3.137*** | 0.323 | 3.128*** | 3.703*** | 7.903*** | 5.746*** | 7.745*** | 5.323*** | 4.302*** |  |
|  | (1.252) | (0.281) | (1.059) | (0.457) | (0.684) | (2.077) | (0.492) | (1.801) | (0.757) | (1.139) |  |
| Lockdown&Pand_cat5 | -1.013 | -2.325*** | 0.387 | -3.283*** | -4.206*** | -6.293*** | -4.876*** | -5.523*** | -6.030*** | -3.252*** |  |
|  | (1.316) | (0.383) | (1.201) | (0.623) | (0.861) | (2.234) | (0.667) | (2.043) | (1.029) | (1.431) |  |
| Constant | 60.758*** | 59.802*** | 63.950*** | 62.931*** | 61.713*** | 53.747*** | 59.051*** | 55.492*** | 52.109*** | 53.243*** |  |
|  | (3.685) | (1.604) | (3.532) | (2.181) | (3.653) | (6.138) | (2.773) | (5.894) | (3.568) | (5.947) |  |
| N | 3,199 | 22,041 | 3,220 | 9,965 | 5,166 | 3,199 | 22,041 | 3,220 | 9,965 | 5,166 |  |
| R^2^ | 0.088 | 0.089 | 0.077 | 0.098 | 0.081 | 0.018 | 0.016 | 0.016 | 0.015 | 0.025 |  |
| F | 11.317 | 70.496 | 10.099 | 45.686 | 18.214 | 1.937 | 12.574 | 1.856 | 5.007 | 4.710 |  |
| p | 0.000 | 0.000 | 0.000 | 0.000 | 0.000 | 0.000 | 0.000 | 0.000 | 0.000 | 0.000 |  |
|  | PHQ-8 Depression Index | | | | | Anxiety Index | | | | | |
| **Income quartile** | Lowest quartile | Second quartile | Third quartile | Highest quartile |  | Lowest quartile | Second quartile | Third quartile | Highest quartile |  |  |
| Lockdown | 0.801** | 1.769*** | 1.507*** | 0.891** |  | 5.097*** | 4.100*** | 3.706*** | 4.526*** |  |  |
|  | (0.461) | (0.404) | (0.411) | (0.412) |  | (0.718) | (0.703) | (0.734) | (0.748) |  |  |
| Pandemic_cat5 | 2.858*** | 1.696*** | 3.922*** | 2.995*** |  | 6.377*** | 4.512*** | 5.751*** | 5.999*** |  |  |
|  | (0.474) | (0.420) | (0.418) | (0.413) |  | (0.737) | (0.730) | (0.746) | (0.750) |  |  |
| Lockdown&Pand_cat5 | -2.436*** | -2.018*** | -3.632*** | -2.112*** |  | -5.797*** | -4.084*** | -4.645*** | -5.157*** |  |  |
|  | (0.608) | (0.545) | (0.541) | (0.539) |  | (0.946) | (0.946) | (0.965) | (0.976) |  |  |
| Constant | 53.647*** | 57.026*** | 53.102*** | 58.399*** |  | 60.163*** | 61.801*** | 62.218*** | 64.131*** |  |  |
|  | (1.533) | (1.835) | (1.816) | (1.836) |  | (2.371) | (3.153) | (3.421) | (3.290) |  |  |
| N | 11,264 | 11,149 | 10,852 | 10,325 |  | 11,264 | 11,149 | 10,852 | 10,325 |  |  |
| R^2^ | 0.077 | 0.063 | 0.078 | 0.079 |  | 0.015 | 0.013 | 0.016 | 0.018 |  |  |
| F | 41.489 | 35.700 | 43.611 | 43.063 |  | 6.056 | 5.815 | 6.741 | 7.492 |  |  |
| p | 0.000 | 0.000 | 0.000 | 0.000 |  | 0.000 | 0.000 | 0.000 | 0.000 |  |  |
|  | PHQ-8 Depression Index | | | | | Anxiety Index | | | | | |
| **Household members** | One | Two | Three | More than 3 |  | One | Two | Three | More than 3 |  |  |
| Lockdown | 1.262*** | 1.103*** | 1.171*** | 1.302*** |  | 5.112*** | 3.313*** | 3.524*** | 5.960*** |  |  |
|  | (0.511) | (0.362) | (0.489) | (0.387) |  | (0.828) | (0.628) | (0.834) | (0.678) |  |  |
| Pandemic_cat5 | 2.571*** | 2.947*** | 2.391*** | 3.202*** |  | 5.908*** | 5.382*** | 5.214*** | 6.610*** |  |  |
|  | (0.545) | (0.364) | (0.499) | 0.389) |  | (0.881) | (0.633) | (0.852) | (0.683) |  |  |
| Lockdown&Pand_cat5 | -2.967*** | -2.528*** | -1.901*** | -2.772*** |  | -4.825*** | -4.421*** | -4.302*** | -6.383*** |  |  |
|  | (0.688) | (0.476) | (0.634) | (0.517) |  | (1.111) | (0.826) | (1.105) | (0.906) |  |  |
| Constant | 57.893*** | 51.253*** | 57.778*** | 52.497*** |  | 66.482*** | 61.747*** | 64.977*** | 61.871*** |  |  |
|  | (1.933) | (1.430) | (2.018) | (1.585) |  | (3.098) | (2.465) | (3.405) | (2.752) |  |  |
| N | 8,566 | 14,929 | 8,445 | 11,651 |  | 8,566 | 14,929 | 8,445 | 11,651 |  |  |
| R^2^ | 0.067 | 0.085 | 0.093 | 0.092 |  | 0.016 | 0.014 | 0.016 | 0.019 |  |  |
| F | 26.670 | 54.045 | 41.986 | 52.942 |  | 5.056 | 8.413 | 5.401 | 7.792 |  |  |
| p | 0.000 | 0.000 | 0.000 | 0.000 |  | 0.000 | 0.000 | 0.000 | 0.000 |  |  |
|  | PHQ-8 Depression Index | | | | | Anxiety Index | | | | | |
| **Region** | Eastern Europe | Northern Europe | Southern Europe |  |  | Eastern Europe | Northern Europe | Southern Europe |  |  |  |
| Lockdown | 0.459*** | 1.074*** | 11.948*** |  |  | 5.385*** | 2.998*** | 8.956*** |  |  |  |
|  | (0.138) | (0.438) | (3.110) |  |  | (1.216) | (0.738) | (2.294) |  |  |  |
| Pandemic_cat5 | 4.390*** | 3.357*** | 13.192*** |  |  | 1.518*** | 7.816*** | 14.781*** |  |  |  |
|  | (1.038) | (0.249) | (3.128) |  |  | (0.646) | (0.420) | (2.324) |  |  |  |
| Lockdown&Pand_cat5 | -4.112*** | -2.932*** | -9.688*** |  |  | -0.341*** | -7.643*** | -9.787*** |  |  |  |
|  | (1.209) | (1.259) | (2.218) |  |  | (0.042) | (3.698) | (2.387) |  |  |  |
| Constant | 58.937*** | 57.644*** | 56.268*** |  |  | 23.085*** | 62.611*** | 67.418*** |  |  |  |
|  | (9.613) | (1.529) | (6.937) |  |  | (14.744) | (2.557) | (11.191) |  |  |  |
| N | 3,328 | 17,333 | 4,497 |  |  | 3,327 | 17,333 | 4,497 |  |  |  |
| R^2^ | 0.071 | 0.093 | 0.110 |  |  | 0.043 | 0.027 | 0.022 |  |  |  |
| F | 8.648 | 65.706 | 22.896 |  |  | 4.962 | 19.691 | 3.651 |  |  |  |
| p | 0.000 | 0.000 | 0.000 |  |  | 0.000 | 0.000 | 0.000 |  |  |  |

Lockdown is a binary variable that takes the value one from the day the lockdown becomes effective, and 0 before.

Pandemic category 5: is a binary variable if the case fatality rate is higher or equal than 2 per cent. The case fatality rate is the percentage of deceased with respect to confirmed cases. The category 5 corresponds to the highest level of the Pandemic Severity Index. <https://www.cdc.gov/media/pdf/mitigationslides.pdf>

Robust standard errors. ***, ** and * denote statistical signiﬁcance at the 1%, 5% and 10% level.

**Table C4. Estimated coefficients for difference-in-difference-in-difference model: effect of lockdown, pandemic category 5 and income quartile.**

|  | Dif-in-dif-in-dif model with lockdown, pandemic cat. 5 and income | |
| --- | --- | --- |
|  | Depression Index | Anxiety  Index |
| Income quartil (4th quartil= highest: omitted) |  |  |
| First (lowest) | 2.822*** | 2.635*** |
|  | (0.506) | (0.171) |
| Second | 4.078*** | -0.314*** |
|  | (0.499) | (0.119) |
| Third | 3.854*** | 0.114*** |
|  | (0.305) | (0.031) |
| Lockdown | 1.247*** | 4.509*** |
|  | (0.227) | (0.397) |
| Pandemic Category 5 | 2.944*** | 5.917*** |
|  | (0.305) | (0.322) |
| Lockdown & Pan_Cat 5 | -2.475*** | -5.143*** |
|  | (0.270) | (0.406) |
| Lockdown & 1st income quartil | 2.563*** | 0.945*** |
|  | (0.311) | (0.332) |
| Lockdown & 2nd income quartil | -1.425*** | 0.262*** |
|  | (0.302) | (0.102) |
| Lockdown & 3rd income quartil | 0.344 | -0.657 |
|  | (0.302) | (0.519) |
| Pan_Cat 5 &1st income quartil | 2.538*** | 1.555*** |
|  | (0.350) | (0.603) |
| Pan_Cat 5 & 2nd income quartil | -0.267 | -0.036 |
|  | (0.346) | (0.596) |
| Pan_Cat 5 & 3rd income quartil | 0.751 | 0.306 |
|  | (0.550) | (0.218) |
| Lockdown & Pan_Cat 5 & 1st income quartil | 2.948*** | 1.577*** |
|  | (0.602) | (0.730) |
| Lockdown & Pan_Cat 5 & 2nd income quartil | -0.948 | -0.588 |
|  | (0.604) | (0.305) |
| Lockdown & Pan_Cat 5 & 3rd income quartil | 1.344 | 0.464 |
|  | (1.629) | (0.268) |
| Constant | 53.722*** | 58.859*** |
|  | (0.875) | (0.296) |
| N | 44,840 | 44,840 |
| R2 | 0.387 | 0.315 |
| F | 116.742 | 16.293 |
| p | 0.000 | 0.000 |

Lockdown is a binary variable that takes the value one from the day the lockdown becomes effective, and 0 before.

Pandemic category 5: is a binary variable if the case fatality rate is higher or equal than 2 per cent. The case fatality rate is the percentage of deceased with respect to confirmed cases. The category 5 corresponds to the highest level of the Pandemic Severity Index. <https://www.cdc.gov/media/pdf/mitigationslides.pdf>

Income quartiles are obtained from adjusted household income before taxes (dividing by the square root of household size).

Change of hour is a binary the value 1 after the hour change (that is, from March 29th onwards) and 0 otherwise.

All regression include gender, age and its squared, being married, years of education, number of household members, having any comorbidity and number of comorbidities, day fixed effects and country fixed effects. Robust standard errors. ***, ** and * denote statistical signiﬁcance at the 1%, 5% and 10% level.

**Table C5. Difference in difference model for PHQ-8 Depression Index and its items. Effect of lockdown and and FDA approval of chloroquine and hydroxychloroquine for clinical patients**

|  | PHQ-8 Depression Index | | | | | Item 1: Little interest or pleasure in doing things | | | | |
| --- | --- | --- | --- | --- | --- | --- | --- | --- | --- | --- |
|  | M1 | M2 | M3 | M4 | M5 | M1 | M2 | M3 | M4 | M5 |
| Lockdown | 1.564*** | 1.270*** | 1.271*** | 1.268*** | 1.283*** | -0.070 | -0.067 | -0.068 | -0.069 | -0.050 |
|  | (0.142) | (0.241) | (0.240) | (0.240) | (0.239) | (0.314) | (0.312) | (0.312) | (0.212) | (0.211) |
| Hydroxychloroquine | 30.138 | 26.279 | 25.457 | 25.313 | 23.252 | 8.679 | 3.146 | 2.470 | 2.545 | -0.575 |
|  | (19.377) | (19.200) | (19.196) | (19.191) | (19.152) | (17.072) | (16.991) | (16.991) | (16.991) | (16.972) |
| Lockdown* Hydroxychloroquine | -0.735 | -0.434 | -0.457 | -0.004 | -0.645 | 0.916 | 1.294 | 1.278 | 1.072 | 1.039 |
|  | (1.130) | (1.117) | (1.117) | (1.121) | (1.115) | (1.717) | (1.705) | (1.705) | (1.711) | (1.703) |
| Constant | 24.807*** | 24.965*** | 24.982*** | 25.000*** | 25.000*** | 24.817*** | 24.984*** | 24.992*** | 24.976*** | 24.997*** |
|  | (0.517) | (0.517) | (0.525) | (0.606) | (0.531) | (0.779) | (0.783) | (0.795) | (0.916) | (0.803) |
| N | 48,434 | 48,434 | 48,434 | 48,434 | 48,434 | 48,434 | 48,434 | 48,434 | 48,434 | 48,434 |
| R^2^ | 0.273 | 0.282 | 0.282 | 0.293 | 0.286 | 0.231 | 0.237 | 0.237 | 0.241 | 0.239 |
| F | 114.357 | 120.095 | 111.208 | 118.575 | 107.865 | 49.961 | 56.379 | 51.947 | 53.556 | 50.728 |
| p | 0.0000 | 0.0000 | 0.0000 | 0.0000 | 0.0000 | 0.0000 | 0.0000 | 0.0000 | 0.0000 | 0.0000 |
|  | Item 2: Feeling down | | | | | Item 3: Trouble falling asleep or sleeping too much | | | | |
|  | M1 | M2 | M3 | M4 | M5 | M1 | M2 | M3 | M4 | M5 |
| Lockdown | -0.211 | -0.198 | -0.196 | -0.101 | -0.184 | 2.956*** | 3.179*** | 3.178*** | 3.186*** | 3.168*** |
|  | (0.202) | (0.201) | (0.200) | (0.201) | (0.200) | (0.327) | (0.324) | (0.322) | (0.320) | (0.320) |
| Hydroxychloroquine | 16.844 | 11.898 | 10.740 | 10.508 | 7.663 | 7.457 | 3.094 | 2.691 | 2.249 | 0.313 |
|  | (26.119) | (25.976) | (25.971) | (25.966) | (25.929) | (17.822) | (17.778) | (17.778) | (17.770) | (17.769) |
| Lockdown* Hydroxychloroquine | -3.786 | -3.471 | -3.498 | -3.620 | -3.717 | 4.871 | 5.193 | 5.182 | 5.173 | 4.990 |
|  | (2.613) | (2.601) | (2.601) | (2.607) | (2.599) | (3.828) | (3.820) | (3.820) | (3.826) | (3.820) |
| Constant | 24.949*** | 24.996*** | 24.984*** | 24.881*** | 24.919*** | 24.994*** | 24.901*** | 24.887*** | 24.474*** | 24.795*** |
|  | (0.733) | (0.736) | (0.747) | (0.861) | (0.755) | (0.827) | (0.833) | (0.847) | (0.975) | (0.857) |
| N | 48,434 | 48,434 | 48,434 | 48,434 | 48,434 | 48,434 | 48,434 | 48,434 | 48,434 | 48,434 |
| R^2^ | 0.256 | 0.261 | 0.261 | 0.266 | 0.264 | 0.231 | 0.235 | 0.235 | 0.242 | 0.237 |
| F | 89.177 | 91.106 | 84.229 | 86.550 | 81.653 | 49.962 | 53.642 | 49.379 | 55.462 | 47.207 |
| p | 0.0000 | 0.0000 | 0.0000 | 0.0000 | 0.0000 | 0.0000 | 0.0000 | 0.0000 | 0.0000 | 0.0000 |
|  | Item 4: Feeling tired or having little energy | | | | | Item 5: Poor appetite or overeating | | | | |
|  | M1 | M2 | M3 | M4 | M5 | M1 | M2 | M3 | M4 | M5 |
| Lockdown | -0.261 | -0.259 | -0.258 | -0.259 | -0.250 | 3.245*** | 2.967*** | 2.968*** | 2.959*** | 2.977*** |
|  | (0.319) | (0.318) | (0.316) | (0.314) | (0.314) | (0.326) | (0.325) | (0.321) | (0.319) | (0.315) |
| Hydroxychloroquine | 43.086 | 40.474 | 39.830 | 39.796 | 37.942 | 54.348 | 51.012 | 50.459 | 50.136 | 49.114 |
|  | (27.520) | (27.458) | (27.456) | (27.457) | (27.431) | (17.214) | (17.138) | (17.137) | (17.125) | (17.128) |
| Lockdown* Hydroxychloroquine | 0.477 | 0.718 | 0.698 | 0.824 | 0.487 | -2.452 | -2.113 | -2.134 | -0.794 | -2.272 |
|  | (1.722) | (1.717) | (1.717) | (1.724) | (1.715) | (1.738) | (1.728) | (1.726) | (1.732) | (1.726) |
| Constant | 24.881*** | 24.706*** | 24.651*** | 24.601*** | 24.487*** | 24.744*** | 24.956*** | 24.974*** | 24.915*** | 24.997*** |
|  | (0.781) | (0.788) | (0.800) | (0.922) | (0.808) | (0.788) | (0.792) | (0.804) | (0.926) | (0.813) |
| N | 48,434 | 48,434 | 48,434 | 48,434 | 48,434 | 48,434 | 48,434 | 48,434 | 48,434 | 48,434 |
| R^2^ | 0.343 | 0.345 | 0.345 | 0.354 | 0.347 | 0.348 | 0.355 | 0.355 | 0.361 | 0.356 |
| F | 69.181 | 68.224 | 62.921 | 71.185 | 60.533 | 76.937 | 82.397 | 75.891 | 79.520 | 71.544 |
| p | 0.0000 | 0.0000 | 0.0000 | 0.0000 | 0.0000 | 0.0000 | 0.0000 | 0.0000 | 0.0000 | 0.0000 |
|  | Item 6: Feeling bad about oneself | | | | | Item 7: Trouble concentrating on things | | | | |
|  | M1 | M2 | M3 | M4 | M5 | M1 | M2 | M3 | M4 | M5 |
| Lockdown | 0.457 | 0.479 | 0.482 | 0.481 | 0.498 | 3.746*** | 3.453*** | 3.454*** | 3.453*** | 3.468*** |
|  | (0.251) | (0.250) | (0.250) | (0.250) | (0.250) | (0.340) | (0.339) | (0.336) | (0.329) | (0.329) |
| Hydroxychloroquine | 25.223 | 20.747 | 18.935 | 18.895 | 15.646 | 44.923 | 40.789 | 40.410 | 40.378 | 38.740) |
|  | (25.110) | (24.966) | (24.950) | (24.950) | (24.894) | (17.452) | (17.369) | (17.368) | (17.368) | (17.359) |
| Lockdown* Hydroxychloroquine | -4.012 | -3.708 | -3.753 | -3.643 | -3.994 | -0.613 | -0.231 | -0.245 | -0.124 | -0.390 |
|  | (2.536) | (2.526) | (2.524) | (2.530) | (2.520) | (1.772) | (1.759) | (1.759) | (1.767) | (1.759) |
| Constant | 23.924*** | 24.423*** | 24.580*** | 24.629*** | 24.780*** | 25.000*** | 24.890*** | 24.870*** | 24.839*** | 24.774*** |
|  | (0.698) | (0.702) | (0.712) | (0.821) | (0.719) | (0.802) | (0.806) | (0.819) | (0.945) | (0.828) |
| N | 48,434 | 48,434 | 48,434 | 48,434 | 48,434 | 48,434 | 48,434 | 48,434 | 48,434 | 48,434 |
| R^2^ | 0.142 | 0.148 | 0.148 | 0.153 | 0.152 | 0.153 | 0.161 | 0.162 | 0.165 | 0.163 |
| F | 68.426 | 72.366 | 66.698 | 69.031 | 66.921 | 84.686 | 91.237 | 85.303 | 84.396 | 80.702 |
| p | 0.0000 | 0.0000 | 0.0000 | 0.0000 | 0.0000 | 0.0000 | 0.0000 | 0.0000 | 0.0000 | 0.0000 |
|  | Item 8: Moving or speaking too slowly or too fidgety | | | | |  | | | | |
|  | M1 | M2 | M3 | M4 | M5 |  |  |  |  |  |
| Lockdown | 1.810*** | 1.716*** | 1.718*** | 1.716*** | 1.725*** |  |  |  |  |  |
|  | (0.225) | (0.229) | (0.233) | (0.234) | (0.234) |  |  |  |  |  |
| Hydroxychloroquine | 29.657 | 28.179 | 27.214 | 27.141 | 25.961 |  |  |  |  |  |
|  | (18.392) | (18.364) | (18.356) | (18.354) | (18.341) |  |  |  |  |  |
| Lockdown* Hydroxychloroquine | 1.480 | 1.603 | 1.574 | 1.520 | 1.451 |  |  |  |  |  |
|  | (1.064) | (1.062) | (1.062) | (1.066) | (1.062) |  |  |  |  |  |
| Constant | 21.919*** | 22.249*** | 22.456*** | 22.697*** | 22.688*** |  |  |  |  |  |
|  | (0.488) | (0.493) | (0.499) | (0.577) | (0.505) |  |  |  |  |  |
| N | 48,434 | 48,434 | 48,434 | 48,434 | 48,434 |  |  |  |  |  |
| R^2^ | 0.119 | 0.120 | 0.120 | 0.127 | 0.122 |  |  |  |  |  |
| F | 31.536 | 31.021 | 28.799 | 35.600 | 28.102 |  |  |  |  |  |
| p | 0.0000 | 0.0000 | 0.0000 | 0.0000 | 0.0000 |  |  |  |  |  |

Lockdown is a binary variable that takes the value one from the day the lockdown becomes effective, and 0 before.

Hydroxychloroquine is a binary variable that takes the value 1 after the FDA approval of chloroquine and hydroxychloroquine for clinical patients (that is, from March 30th onwards) for country c and day t, and 0 before.

M1 includes lockdown, pandemic of category 5, interaction between lockdown and pandemic of category 5, day fixed effects and country fixed effects. M2 includes the same explanatory variables than M1 and also male, other gender (omitted: women), age and its squared. M3 includes the same explanatory variables than M2 and also married (omitted: single), years of education and number of household members (omitted: living alone). M4 includes the same explanatory variables than M3 and also having any comorbidity and number of comorbidities. M5 includes the same explanatory variables than M4 and also household income quartile (omitted: lowest quartile). Individual sample weights have been used to correct for differences in income, education, age and gender structure between the general population of the country and the corresponding sample. Robust standard errors. ***, ** and * denote statistical signiﬁcance at the 1%, 5% and 10% level.

**Table C6. Difference in difference model for Anxiety Index and its items. Effect of lockdown and FDA approval of chloroquine and hydroxychloroquine for clinical patients**

|  | Anxiety Index | | | | | Item 1: Nervous when thinking about current circumstances | | | | | |  |
| --- | --- | --- | --- | --- | --- | --- | --- | --- | --- | --- | --- | --- |
|  | M1 | M2 | M3 | M4 | M5 | | M1 | M2 | M3 | M4 | M5 | |
| Lockdown | 4.398*** | 4.414*** | 4.415*** | 4.406*** | 4.417*** | | -0.527 | -0.560 | -0.558 | -0.576 | -0.556 | |
|  | (0.339) | (0.338) | (0.337) | (0.336) | (0.336) | | (0.327) | (0.324) | (0.324) | (0.320) | (0.321) | |
| Hydroxychloroquine | -6.024 | -5.676 | -6.267 | -6.802 | -7.091 | | -7.388 | -6.062 | -7.208 | -8.146 | -7.462 | |
|  | (29.695) | (29.695) | (29.695) | (29.676) | (29.694) | | (17.482) | (17.479) | (17.477) | (17.444) | (17.477) | |
| Lockdown* Hydroxychloroquine | -3.900*** | -3.875*** | -3.860*** | -3.830*** | -3.775*** | | 0.511 | 0.430 | 0.402 | 2.885 | 0.412 | |
|  | (1.482) | (1.482) | (1.482) | (1.487) | (1.482) | | (1.776) | (1.776) | (1.776) | (1.778) | (1.776) | |
| Constant | 22.944*** | 22.994*** | 22.907*** | 21.410*** | 22.793*** | | 18.837*** | 19.165*** | 18.878*** | 14.412*** | 18.823*** | |
|  | (0.859) | (0.868) | (0.881) | (0.887) | (0.892) | | (0.804) | (0.813) | (0.826) | (0.951) | (0.836) | |
| N | 48,434 | 48,434 | 48,434 | 48,434 | 48,434 | | 48,434 | 48,434 | 48,434 | 48,434 | 48,434 | |
| R^2^ | 0.213 | 0.214 | 0.214 | 0.217 | 0.214 | | 0.272 | 0.273 | 0.273 | 0.274 | 0.274 | |
| F | 22.010 | 21.132 | 19.467 | 22.056 | 18.213 | | 112.205 | 107.874 | 100.125 | 96.156 | 93.138 | |
| p | 0.0000 | 0.0000 | 0.0000 | 0.0000 | 0.0000 | | 0.0000 | 0.0000 | 0.0000 | 0.0000 | 0.0000 | |
|  | Item 2: Worried about one’s health | | | | | Item 3: Worried about family’s health | | | | | |  |
|  | M1 | M2 | M3 | M4 | M5 | | M1 | M2 | M3 | M4 | M5 | |
| Lockdown | 0.989*** | 0.930*** | 0.930*** | 0.927*** | 0.929*** | | 4.354*** | 4.416*** | 4.416*** | 4.408*** | 4.418*** | |
|  | (0.327) | (0.321) | (0.317) | (0.317) | (0.317) | | (0.336) | (0.329) | (0.328) | (0.326) | (0.326) | |
| Hydroxychloroquine | -4.195 | -4.607 | -4.733 | -4.261 | -4.137 | | 1.309 | 2.573 | 2.636 | 2.210 | 1.973 | |
|  | (7.808) | (7.803) | (7.804) | (7.793) | (7.803) | | (10.772) | (10.769) | (10.769) | (10.762) | (10.769) | |
| Lockdown* Hydroxychloroquine | -5.048*** | -4.984*** | -4.985*** | -5.004*** | -4.968*** | | -2.686*** | -2.767*** | -2.765*** | 2.380*** | -2.806*** | |
|  | (1.744) | (1.744) | (1.744) | (1.751) | (1.744) | | (0.820) | (0.820) | (0.820) | (0.824) | (0.820) | |
| Constant | 24.603*** | 24.523*** | 24.515*** | 24.868*** | 24.545*** | | 23.603*** | 23.757*** | 23.765*** | 22.788*** | 23.692*** | |
|  | (0.791) | (0.800) | (0.812) | (0.936) | (0.822) | | (0.823) | (0.833) | (0.846) | (0.974) | (0.857) | |
| N | 48,434 | 48,434 | 48,434 | 48,434 | 48,434 | | 48,434 | 48,434 | 48,434 | 48,434 | 48,434 | |
| R^2^ | 0.210 | 0.211 | 0.211 | 0.212 | 0.211 | | 0.211 | 0.211 | 0.211 | 0.222 | 0.212 | |
| F | 16.913 | 16.457 | 15.122 | 15.542 | 14.150 | | 18.367 | 17.641 | 16.275 | 29.612 | 15.224 | |
| p | 0.0000 | 0.0000 | 0.0000 | 0.0000 | 0.0000 | | 0.0000 | 0.0000 | 0.0000 | 0.0000 | 0.0000 | |
|  | Item 4: Stressed about leaving the house | | | | | |  | | | | | |
|  | M1 | M2 | M3 | M4 | M5 | |  |  |  |  |  |  |
| Lockdown | 5.021*** | 5.033*** | 5.031*** | 5.049*** | 5.030*** | |  |  |  |  |  |  |
|  | (0.357) | (0.357) | (0.356) | (0.353) | (0.356) | |  |  |  |  |  |  |
| Hydroxychloroquine | -3.229 | -3.518 | -3.168 | -3.436 | -3.288 | |  |  |  |  |  |  |
|  | (8.415) | (8.415) | (8.384) | (8.328) | (8.384) | |  |  |  |  |  |  |
| Lockdown* Hydroxychloroquine | -2.655*** | -2.641*** | -2.672*** | -2.534*** | -2.680*** | |  |  |  |  |  |  |
|  | (0.455) | (0.455) | (0.453) | (0.428) | (0.453) | |  |  |  |  |  |  |
| Constant | 16.855*** | 16.782*** | 16.358*** | 10.176*** | 16.328*** | |  |  |  |  |  |  |
|  | (0.211) | (0.213) | (0.216) | (0.234) | (0.218) | |  |  |  |  |  |  |
| N | 48,434 | 48,434 | 48,434 | 48,434 | 48,434 | |  |  |  |  |  |  |
| R^2^ | 0.246 | 0.246 | 0.247 | 0.250 | 0.248 | |  |  |  |  |  |  |
| F | 73.403 | 69.203 | 65.585 | 65.755 | 61.378 | |  |  |  |  |  |  |
| p | 0.0000 | 0.0000 | 0.0000 | 0.0000 | 0.0000 | |  |  |  |  |  |  |

Lockdown is a binary variable that takes the value one from the day the lockdown becomes effective, and 0 before.

Hydroxychloroquine is a binary variable that takes the value 1 after the FDA approval of chloroquine and hydroxychloroquine or clinical patients (that is, from March 30th onwards) for country c and day t, and 0 before.

M1 includes lockdown, pandemic of category 5, interaction between lockdown and pandemic of category 5, day fixed effects and country fixed effects. M2 includes the same explanatory variables than M1 and also male, other gender (omitted: women), age and its squared. M3 includes the same explanatory variables than M2 and also married (omitted: single), years of education and number of household members (omitted: living alone). M4 includes the same explanatory variables than M3 and also having any comorbidity and number of comorbidities. M5 includes the same explanatory variables than M4 and also household income quartile (omitted: lowest quartile). Individual sample weights have been used to correct for differences in income, education, age and gender structure between the general population of the country and the corresponding sample. Robust standard errors. ***, ** and * denote statistical signiﬁcance at the 1%, 5% and 10% level.

**Appendix D**

**Figure D1. McCrary density function of daily responses to the online survey**

|  |  |
| --- | --- |
|  |  |

Note: The vertical axis shows the McCrary density function of the days elapsed since lockdown became effective (upper figures) or days elapsed since pandemic reached category 5 (lower figures). The white circles are the mean of the dependent variable for a given value of days before/after lockdown (or pandemic reached category 5). The black lines are the predicted probabilities for the outcome variable on the treatment variable and a quadratic polynomial in terms of days before/after cut-off point. The red vertical line indicates the day when lockdown became effective or when pandemic reached category 5. Therefore, negative values on the horizontal axis denote interviews performed before lockdown became effective (or before pandemic reached category 5), whereas positive values on the horizontal axis denote interviews after lockdown became effective (or after pandemic reached category 5).

**Table D1. RD design. Difference in regression discontinuity. Items of the PHQ-Depression Index**

|  | MSE optimal | CER optimal | Without covariates | Alternative bandwidth | | False threshold | |
| --- | --- | --- | --- | --- | --- | --- | --- |
|  |  |  |  | 6 DAYS | 4 DAYS | 2 days before | 2 days after |
| **Running variable: Days elapsed since lockdown** |  |  |  |  |  |  |  |
| Item 1: Little interest or pleasure in doing things |  |  |  |  |  |  |  |
| Lockdown | -0.035 | -0.036 | -0.034 | -0.033 | -0.033 | -0.038 | -0.039 |
|  | (0.298) | (0.304) | (0.289) | (0.280) | (0.275) | (0.366) | (0.373) |
| Lockdown*Pan_cat5 | -0.544 | -0.555 | -0.528 | -0.512 | -0.502 | -0.588 | -0.599 |
|  | (0.394) | (0.402) | (0.381) | (0.370) | (0.363) | (4.173) | (4.261) |
| Item 2: Feeling down |  |  |  |  |  |  |  |
| Lockdown | -0.112 | -0.114 | -0.109 | -0.105 | -0.103 | -0.121 | -0.123 |
|  | (0.278) | (0.283) | (0.269) | (0.261) | (0.256) | (0.341) | (0.348) |
| Lockdown*Pan_cat5 | -1.826*** | -1.863*** | -1.771*** | -1.718*** | -1.684*** | -1.971 | -2.011 |
|  | (0.367) | (0.374) | (0.356) | (0.345) | (0.338) | (3.882) | (3.963) |
| Item 3: Trouble falling asleep or sleeping too much |  |  |  |  |  |  |  |
| Lockdown | 3.016*** | 3.078*** | 2.923*** | 2.833*** | 2.775*** | 3.265 | 3.332 |
|  | (0.319) | (0.325) | (0.309) | (0.300) | (0.294) | (2.448) | (2.456) |
| Lockdown*Pan_cat5 | -5.297*** | -5.402*** | -5.139*** | -4.986*** | -4.886*** | -5.718 | -5.832 |
|  | (0.422) | (0.431) | (0.410) | (0.398) | (0.390) | (4.486) | (4.579) |
| Item 4: Feeling tired or having little energy |  |  |  |  |  |  |  |
| Lockdown | -0.279 | -0.285 | -0.271 | -0.263 | -0.257 | -0.302 | -0.308 |
|  | (0.299) | (0.305) | (0.290) | (0.281) | (0.275) | (0.367) | (0.374) |
| Lockdown*Pan_cat5 | -2.254*** | -2.299*** | -2.186*** | -2.120*** | -2.079*** | -2.433 | -2.482 |
|  | (0.396) | (0.404) | (0.383) | (0.372) | (0.364) | (4.193) | (4.281) |
| Item 5: Poor appetite or overeating |  |  |  |  |  |  |  |
| Lockdown | 1.678*** | 1.712*** | 1.627*** | 1.577*** | 1.545*** | 1.815 | 1.851 |
|  | (0.301) | (0.307) | (0.292) | (0.284) | (0.278) | (0.370) | (0.378) |
| Lockdown*Pan_cat5 | -2.385*** | -2.433*** | -2.315*** | -2.245*** | -2.200*** | -2.576 | -2.627 |
|  | (0.399) | (0.407) | (0.386) | (0.374) | (0.367) | (4.224) | (4.311) |
| Item 6: Feeling bad about oneself |  |  |  |  |  |  |  |
| Lockdown | 0.472** | 0.481** | 0.457** | 0.444** | 0.435** | 0.510 | 0.520 |
|  | (0.215) | (0.210) | (0.217) | (0.219) | (0.214) | (0.326) | 0.332) |
| Lockdown*Pan_cat5 | -1.436*** | -1.465*** | -1.393*** | -1.351*** | -1.324*** | -1.551 | -1.581 |
|  | (0.350 | (0.357) | (0.340) | (0.330) | (0.323) | (3.703) | (3.779) |
| Item 7: Trouble concentrating on things |  |  |  |  |  |  |  |
| Lockdown | 3.272*** | 3.340*** | 3.171*** | 3.073*** | 3.009*** | 3.542 | 3.616 |
|  | (0.304) | (0.310) | (0.295) | (0.286) | (0.280) | (2.374) | (2.381) |
| Lockdown*Pan_cat5 | -4.435*** | -4.523*** | -4.303*** | -4.174*** | -4.091*** | -4.789 | -4.884 |
|  | (0.403) | (0.411) | (0.391) | (0.379) | (0.371) | (4.274) | 4.363) |
| Item 8: Moving or speaking too slowly or too fidgety |  |  |  |  |  |  |  |
| Lockdown | 1.152*** | 1.176*** | 1.117*** | 1.083*** | 1.062*** | 1.245 | 1.271 |
|  | (0.187) | (0.190) | (0.181) | (0.176) | (0.172) | (2.230) | (1.235) |
| Lockdown*Pan_cat5 | -1.156*** | -1.179*** | -1.121*** | -1.087*** | -1.065*** | -1.247 | -1.272 |
|  | (0.249) | (0.253) | (0.241) | (0.234) | (0.229) | (2.596) | (2.650) |
| **Running variable: Days elapsed since pandemic reached category 5** |  |  |  |  |  |  |  |
| Item 1: Little interest or pleasure in doing things |  |  |  |  |  |  |  |
| Pan_cat5 | -0.928*** | -0.947*** | -0.901*** | -0.874*** | -0.857*** | -1.002 | -1.022 |
|  | (0.303) | (0.309) | (0.294) | (0.285) | (0.280) | (3.187) | (3.252) |
| Lockdown*Pan_cat5 | -0.445 | -0.454 | -0.432 | -0.419 | -0.411 | -0.481 | -0.490 |
|  | (0.322) | (0.328) | (0.312) | (0.303) | (0.297) | (3.391) | (3.460) |
| Item 2: Feeling down |  |  |  |  |  |  |  |
| Pan_cat5 | 3.088*** | 3.150*** | 2.993*** | 2.900*** | 2.840*** | 3.342 | 3.411 |
|  | (0.283) | (0.289) | (0.275) | (0.266) | (0.261) | (2.969) | (3.031) |
| Lockdown*Pan_cat5 | -1.495*** | -1.524*** | -1.450*** | -1.406*** | -1.378*** | -1.613 | -1.646 |
|  | (0.300) | (0.306) | (0.291) | (0.283) | (0.277) | (3.156) | (3.221) |
| Item 3: Trouble falling asleep or sleeping too much |  |  |  |  |  |  |  |
| Pan_cat5 | 5.460*** | 5.574*** | 5.289*** | 5.123*** | 5.015*** | 5.920 | 6.045 |
|  | (0.324) | (0.331) | (0.314) | (0.305) | (0.299) | (3.414) | (3.485) |
| Lockdown*Pan_cat5 | -4.338*** | -4.424*** | -4.208*** | -4.083*** | -4.002*** | -4.684 | -4.777 |
|  | (0.345) | (0.352) | (0.335) | (0.325) | (0.318) | (3.643) | (3.718) |
| Item 4: Feeling tired or having little energy |  |  |  |  |  |  |  |
| Pan_cat5 | 0.771*** | 0.787*** | 0.748*** | 0.725*** | 0.711*** | 0.834 | 0.850 |
|  | (0.304) | (0.310) | (0.295) | (0.286) | (0.280) | (3.196) | (3.262) |
| Lockdown*Pan_cat5 | -1.845*** | -1.881*** | -1.790*** | -1.736*** | -1.701*** | -1.992 | -2.032 |
|  | (0.323) | (0.330) | (0.314) | (0.304) | (0.298) | (3.407) | (3.477) |
| Item 5: Poor appetite or overeating |  |  |  |  |  |  |  |
| Pan_cat5 | 3.033*** | 3.095*** | 2.939*** | 2.849*** | 2.790*** | 3.282 | 3.350 |
|  | (0.307) | (0.313) | (0.298) | (0.289) | (0.283) | (3.226) | (3.292) |
| Lockdown*Pan_cat5 | -1.953*** | -1.992*** | -1.894*** | -1.838*** | -1.801*** | -2.109 | -2.150 |
|  | (0.326) | (0.332) | (0.316) | (0.306) | (0.300) | (3.431) | (3.502) |
| Item 6: Feeling bad about oneself |  |  |  |  |  |  |  |
| Pan_cat5 | 2.808*** | 2.865*** | 2.721*** | 2.637*** | 2.584*** | 3.038 | 3.101 |
|  | (0.270) | (0.276) | (0.262) | (0.254) | (0.249) | (2.832) | (2.890) |
| Lockdown*Pan_cat5 | -1.175*** | -1.199*** | -1.140*** | -1.106*** | -1.084*** | -1.268 | -1.294 |
|  | (0.287) | (0.293) | (0.278) | (0.270) | (0.265) | (3.011) | (3.072) |
| Item 7: Trouble concentrating on things |  |  |  |  |  |  |  |
| Pan_cat5 | 5.905*** | 6.029*** | 5.718*** | 5.538*** | 5.422*** | 6.404 | 6.539 |
|  | (0.310 | (0.317) | (0.301) | (0.292) | (0.286) | (6.651) | (6.792) |
| Lockdown*Pan_cat5 | -3.632*** | -3.704*** | -3.523*** | -3.418*** | -3.350*** | -3.922 | -4.000 |
|  | (0.329) | (0.336) | (0.319) | (0.310) | (0.304) | (3.472) | (3.543) |
| Item 8: Moving or speaking too slowly or too fidgety |  |  |  |  |  |  |  |
| Pan_cat5 | 1.316*** | 1.343*** | 1.276*** | 1.238*** | 1.212*** | 1.423 | 1.453 |
|  | (0.190) | (0.194) | (0.184) | (0.179) | (0.175) | (1.984) | (2.024) |
| Lockdown*Pan_cat5 | -0.945*** | -0.964*** | -0.917*** | -0.889*** | -0.872*** | -1.021 | -1.041 |
|  | (0.203) | (0.207) | (0.196) | (0.191) | (0.187) | (2.115) | (2.158) |

Mean square error (MSE): optimal bandwidth is estimated by taking the minimum optimal bandwidth of the most common MSE-optimal procedures. Coverage error (CER): optimal bandwidth is the minimum bandwidth of the diﬀerent coverage error procedures following Calonico et al. (2018). Optimal bandwidth= 5 days. Robust standard errors. ***, ** and * denote statistical signiﬁcance at the 1%, 5% and 10% level.

**Table D2. RD design. Difference in regression discontinuity. Items of the Anxiety Index**

|  | MSE optimal | CER optimal | Without covariates | Alternative bandwidth | | False threshold | |
| --- | --- | --- | --- | --- | --- | --- | --- |
|  |  |  |  | 6 DAYS | 4 DAYS | 2 days before | 2 days after |
| **Running variable: Days elapsed since lockdown** |  |  |  |  |  |  |  |
| Item 1: Nervous when thinking about current circumstances |  |  |  |  |  |  |  |
| Lockdown | -0.380 | -0.387 | -0.369 | -0.358 | -0.351 | -0.410 | -0.418 |
|  | (0.308) | (0.314) | (0.298) | (0.290) | (0.284) | (0.378) | (0.386) |
| Lockdown*Pan_cat5 | -6.921*** | -7.058*** | -6.715*** | -6.515*** | -6.386*** | -7.470 | -7.619 |
|  | (0.408) | (0.416) | (0.396) | (0.383) | (0.375) | (4.324) | (4.414) |
| Item 2: Worried about one’s health |  |  |  |  |  |  |  |
| Lockdown | 0.764*** | 0.779*** | 0.740*** | 0.718*** | 0.704*** | 0.826 | 0.842 |
|  | (0.307) | (0.313) | (0.298) | (0.289) | (0.283) | (0.577) | (0.585) |
| Lockdown*Pan_cat5 | 1.068*** | 1.089*** | 1.036*** | 1.004*** | 0.984*** | 1.153 | 1.176 |
|  | (0.407) | (0.415) | (0.395) | (0.382) | (0.374) | (4.315) | (4.404) |
| Item 3: Worried about family’s health |  |  |  |  |  |  |  |
| Lockdown | 3.861*** | 3.940*** | 3.740*** | 3.625*** | 3.550*** | 4.182 | 4.268 |
|  | (0.319) | (0.325) | (0.309) | (0.300) | (0.294) | (3.355) | (3.424) |
| Lockdown*Pan_cat5 | -3.785*** | -3.860*** | -3.671*** | -3.562*** | -3.491*** | -4.086 | -4.167 |
|  | (0.422) | (0.431) | (0.410) | (0.398) | (0.390) | (4.486) | (4.579) |
| Item 4: Stressed about leaving the house |  |  |  |  |  |  |  |
| Lockdown | 4.929*** | 5.033*** | 4.774*** | 4.625*** | 4.529*** | 5.342 | 5.455 |
|  | (0.304) | (0.310) | (0.295) | (0.286) | (0.280) | (3.196) | (3.262) |
| Lockdown*Pan_cat5 | -6.846*** | -6.983*** | -6.642*** | -6.445*** | -6.317*** | -8.408 | -8.574 |
|  | (0.402) | (0.410) | (0.390) | (0.378) | (0.370) | (4.964) | )4.353) |
| **Running variable: Days elapsed since pandemic reached category 5** |  |  |  |  |  |  |  |
| Item 1: Nervous when thinking about current circumstances |  |  |  |  |  |  |  |
| Pan_cat5 | 11.513*** | 11.765*** | 11.136*** | 10.772*** | 10.538*** | 12.528 | 12.804 |
|  | (0.314) | (0.320) | (0.305) | (0.296) | (0.290) | (31.174) | (32.106) |
| Lockdown*Pan_cat5 | -5.669*** | -5.783*** | -5.500*** | -5.336*** | -5.230*** | -6.121 | -6.243* |
|  | (0.333) | (0.340) | (0.323) | (0.313) | (0.307) | (3.512) | (3.585) |
| Item 2: Worried about one’s health |  |  |  |  |  |  |  |
| Pan_cat5 | -1.342*** | -1.370*** | -1.304*** | -1.265*** | -1.239*** | -1.448 | -1.478 |
|  | (0.313) | (0.319) | (0.304) | (0.295) | (0.289) | (3.295) | (3.363) |
| Lockdown*Pan_cat5 | 0.874*** | 0.891*** | 0.847*** | 0.822*** | 0.806*** | 0.943 | 0.962 |
|  | (0.332) | (0.339) | (0.322) | (0.313) | (0.307) | (3.504) | (3.577) |
| Item 3: Worried about family’s health |  |  |  |  |  |  |  |
| Pan_cat5 | 5.439*** | 5.554*** | 5.269*** | 5.104*** | 4.996*** | 5.898 | 6.023 |
|  | (0.325) | (0.331) | (0.315) | (0.306) | (0.300) | (3.424) | (3.494) |
| Lockdown*Pan_cat5 | -3.098*** | -3.161*** | -3.006*** | -2.916*** | -2.858*** | -3.346 | -3.412 |
|  | (0.345) | (0.352) | (0.335) | (0.325) | (0.318) | (3.643) | (3.718) |
| Item 4: Stressed about leaving the house |  |  |  |  |  |  |  |
| Pan_cat5 | 10.129*** | 10.334*** | 9.822*** | 9.525*** | 9.333*** | 12.487 | 12.739 |
|  | (0.309) | (0.316) | (0.300) | (0.291) | (0.285) | (7.668) | (7.933) |
| Lockdown*Pan_cat5 | -5.609*** | -5.720*** | -5.441*** | -5.279*** | -5.174*** | -6.890 | -7.027 |
|  | (0.329) | (0.335) | (0.319) | (0.309) | (0.303) | (5.637) | (5.712) |

Mean square error (MSE): optimal bandwidth is estimated by taking the minimum optimal bandwidth of the most common MSE-optimal procedures. Coverage error (CER): optimal bandwidth is the minimum bandwidth of the diﬀerent coverage error procedures following Calonico et al. (2018). Optimal bandwidth= 5 days. Robust standard errors. ***, ** and * denote statistical signiﬁcance at the 1%, 5% and 10% level.
